# Effect of providing gender equality information on students’ motivations to choose STEM

**DOI:** 10.1371/journal.pone.0252710

**Published:** 2021-06-23

**Authors:** Yuko Ikkatai, Atsushi Inoue, Azusa Minamizaki, Kei Kano, Euan McKay, Hiromi M. Yokoyama

**Affiliations:** 1 Kavli Institute for the Physics and Mathematics of the Universe, The University of Tokyo, Kashiwa, Japan; 2 Nippon Institute for Research Advancement, Tokyo, Japan; 3 Kobayashi-Maskawa Institute for the Origin of Particles and the Universe, Nagoya University, Nagoya, Japan; 4 Graduate School of Education, Shiga University, O-tsu, Japan; 5 Division for Strategic Public Relations, The University of Tokyo, Tokyo, Japan; University of Westminster, UNITED KINGDOM

## Abstract

The social climate for women studying STEM subjects is changing, but the proportion of women taking STEM subjects in Japan is small. Only 27.9% of university students in the department of science is women in 2019. In this study, we used an online survey to investigate whether randomly providing three types of gender equality information increased the motivation of junior high school students to choose STEM subjects and the motivation of their parents to support that choice. Information on STEM, especially about social equality, and information on math stereotypes and STEM occupations, increased students’ motivations for studying STEM. This suggests that providing gender equality information is an effective way to change students’ attitudes toward STEM.

## Introduction

### Changing attitudes by providing information

Information stimuli can influence attitudes. The phenomenon of being unconsciously exposed to certain stimuli that influence a subsequent response is called behavioral priming or the priming effect (among others, [1, 2]). Exposure to science, technology, engineering and mathematics (STEM) information changed motivations towards STEM [3–7].

A study targeted upper secondary school students and followed them for five years in Germany investigated whether providing income information changed high school students’ choice of field of study [7]. For the experimental group, they gave a 20-minute presentation on income information that included the relationship between a major subject of study and later income as well as a one-page flyer with general information on college attendance and a list of websites for financial aid. The control group received only the one-page flyer. The results showed that providing income information significantly affected only male student’s choices of fields of study. Male students in the experimental group chose majors leading to better-paid careers, but this trend was not observed in female students [7].

Another study conducted for high school students in the US reported that a belief in success from studying science subjects increased after reading a scientist’s story that included failures and struggles more than another story without failures and struggles [4]. However, the study described this small effect as a limitation of their study and discussed the need to improve the design of intervention [4]. Another study investigated whether virtual experiences of STEM or non-STEM jobs changed female undergraduate students’ interest in STEM courses [6]. Their research found that students who experienced STEM jobs (computer engineer and programmer) using virtual reality (VR) had increased levels of interest, although this effect was limited to participants who identified with the virtual character they were assigned in the study. The increase of interest was not found in students who virtually experienced non-STEM jobs (writer and editor) [6]. Additionally, a study [8] showed that information focusing on gender similarities more than gender differences encouraged female university students to participate in STEM.

These studies suggest that providing STEM information could change students’ motivations for studying STEM subjects, but the effectiveness could depend on the content and the way it was provided. Although there are many practical initiatives to provide STEM information for girls, no quantitative study in Japan has examined whether providing this type of information changes students’ motivations for studying STEM subjects.

### Social climate for women and STEM in Japan

The social climate surrounding women and STEM is changing in Japan. First, people working in STEM careers in mechanical, electronical, civil engineering and information technology are in high demand [9]. The Japanese government and companies encourage girls and women to study and pursue jobs in STEM. Second, the Act on Promotion of Women’s Participation and Advancement in the Workplace was passed in 2016. This act requires employers with over 300 regular workers to analyze several gender issues, such as the female ratio among all employees and managers, as well as their working hours, and report this information to the government [10]. As a result, the percentage of women in management positions increased in several STEM-related companies that changed their internal systems [11].

However, many Japanese people still have an image that science fields are more suitable for men than women [12]. In many Japanese high schools, students need to choose the science (*rikei*) or the humanities (*bunkei*) stream during high school. This choice determines their future choice of courses at the university level. Students choosing the science stream take advanced science classes: chemistry, physics, biology, and earth science. Some students change their stream at high school from science to humanities or from humanities to the science stream. One survey reported that 5.8% of students changed from humanities to the science stream and that 8.5% changed from science to the humanities stream [13], suggesting that these cases are limited. In other words, students’ STEM careers are likely determined before high school. Junior high school students take science classes that include chemistry, physics, biology, and earth science. Several studies have shown that the gender gap in the preference for science widens at junior high school [14, 15]. A study asked 630 Japanese junior high school students to answer how much they liked science subject by 5-point scale from strongly like (= 5) to strongly dislike (= 1). Both boys and girls rated 3.96 on average at the beginning of the first year, but the rating decreased to 3.48 in boys and 2.89 in girls at the third year. Boys significantly rated higher than girls at both second and third year [15]. Today in Japan, gender differences in academic performance in the sciences are rare. One study, Trends in International Mathematics and Science Study (TIMSS in 2015), measured the academic ability of primary school students (fourth year) and junior high school students (second year), finding that there were no significant gender differences in the scores for science [16, 17]. Also, in the Program for International Student Assessment (PISA in 2018) measuring the science literacy of 15-year-old students (first-year high school students in Japan), the scores for sciences were the same for boys and girls [18]. Nevertheless, fewer females are studying STEM subjects at the university level.

### What factors influence women’s choice of STEM?

We focused on three factors based on a literature survey.

#### Availability of STEM occupations

Engineering and information science are two disciplines with strong growth potential [19]. Math-intensive occupations, such as mathematician, statistician, and physicist, also get high salaries [20]. The mean annual salary was higher in STEM jobs (95,350 USD) than all jobs (53,490 USD) in the US in 2019 [21]. Women in STEM jobs earn 33% more than women in non-STEM jobs. However, men still earn 14% more than women in the same STEM jobs [22]. In Japan, according to the results of the Basic Survey on Wage Structure in 2019 conducted by the Ministry of Health, Labour and Welfare, women account for about 24% of natural science researchers. The average monthly salary of natural science researchers (excluding overtime pay) is 422,000 yen, higher than the average for all occupations of 308,000 yen, but there is a large gap between that of male natural science researchers, 444,000 yen, and that of female natural science researchers, 355,000 yen [23]. In the US, women dominate close to half of all jobs. However, only 25% of women worked in STEM jobs in 2009. It has been reported that there are various possible reasons why women leave STEM careers: a lack of female role models, gender stereotyping, and less family-friendly flexibility [22].

#### Women’s underrepresentation in education

Japan is a gender unequal country. The ranking of Japan in the Global Gender Gap Index based on health, education, economy, and politics was 121 out of 153 countries in 2019 [24]. Especially in higher education, the percentage of students in Japanese universities at the bachelor’s level in 2019 was 45.4% women and 54.6% men [25]. In other words, girls and women enter university in smaller numbers than boys and men. The percentages of the Japanese people over 16 years old who wanted a boy to get a university education was 72%, which was higher than that of 61% in girl [26], demonstrating that some people still consider that university education was more important for men than women. The gender gap still exists in Japanese society. Additionally, most STEM fields are dominated by men. The percentage of female university students studying the humanities in 2019 was 65.3%, with even fewer taking STEM subjects: 27.9% in the department of science and 15.4% in engineering. The percentage is also extremely low in mathematics (19.1%), physics (15.5%), and mechanical engineering (5.9%), in contrast to biology (38.8%), and chemistry (31.6%) [27].

Conventionally, Japanese women studying at university, especially STEM subjects, are likely to be recognized as intellectual. Some Japanese people still have a belief that intellectual women are not preferred in Japanese society. Several cases were reported where mothers disagreed with their daughters studying STEM. There was a mother who considered that this would prevent her daughter from getting married [28]. A study pointed out that the level of educational attainment for Japanese women can traditionally be used as an expression of the particular culture of the middle class: females were expected to be a good wife and a wise mother. This view was reflected in the low number of women in professional courses [29]. Parental belief also influences their children’s’ choice in science [30, 31]. Especially, the mother’s belief for her daughter’s success in STEM is related to her daughter’s future STEM career. This suggests that reducing the parents’ gender stereotyping would contribute to increasing the number of girls taking STEM.

For years, many initiatives to encourage girls to study STEM have been conducted at various levels. For example, a campaign called Rico-Challenge (*rico* means STEM in Japanese) targeting junior high school girls or high school girls has been hosted by the Gender Equality Bureau Cabinet Office of the Japanese government [32]. This campaign provides STEM information on the Internet, for example, messages from women in STEM and several offline events that introduce STEM-related occupations to girls. The Japan Science and Technology Agency (JST) financially supports universities and research institutions that have programs for girls to study STEM [33]. A Japanese publisher, Kodansha Ltd., has launched a website called Rikejo (science girls) that provides a range of information on STEM for girls, for example, a method to study science subjects, messages from STEM workers, a science column, and STEM events [34]. Google’s Mind the Gap program provides seminars and workshops on computer science for girls, with a version for Japanese girls [35]. It is assumed that these programs have directly or indirectly contributed to the increase in the number of girls studying STEM. The percentage of female university students increased from 18.3% in 1989 to 27.9% in 2019 in the department of science, and from 3.4% in 1989 to 15.4% in 2019 in the department of engineering [27, 36].

However, whether and how providing STEM information motivates girls to choose STEM for their future careers has yet to be investigated quantitatively.

#### Stereotype threat of math

We posit that at least three stereotyping images and beliefs may prevent girls from studying STEM subjects. The first stereotyping image is that women by nature are not as good at mathematics as men. In Japan, the population of female university students is especially low in mathematics, physics, and engineering [27], which are generally considered to be math-intensive fields [37]. Although the results are inconsistent, several findings suggest that the gender difference in mathematics is lessening [16–18]. Regarding Japanese students, there was no significant gender difference in the scores for mathematics in TIMSS 2015 [16, 17]. On the other hand, in PISA 2018, a difference was reported in math literacy: boys scored higher (532 points) than girls (522 points). The 10-point difference was significantly higher than the world average of 5 points [18]. TIMSS targets primary and junior high school students, but PISA targets high school students. The difference of results between TIMSS and PISA might be due to the differences in the students’ grade level.

However, “stereotype threats” have been reported, or that negative stereotypes of the group that a person belongs to prevent him/her from reaching their full potential. One study showed that females, before taking a math test, scored the same as males if they read a text statement stating that there is no gender difference in math [38]. It is likely that girls who have a gender stereotype that girls are not good at mathematics score lower than boys in math tests. The gender gap in students’ performance varies among countries, and gender gaps are not considered to be innate [39].

## Research questions

We considered that the junior high school period is important for providing STEM information to students. Many STEM events for junior high and high school students have introduced “STEM occupations” in addition to interesting science. However, the information of “gender equality in society” or “girls are good at math,” which we consider important for their motivation for STEM, are rarely shown at many STEM events.

In this study, we investigated whether the gender equality information provided related to the listing of the factors, increased the motivation of junior high school students to choose STEM and the motivation of their parents to support their children. We also investigated whether the gender equality information being provided changed certain perceptions on gender equality, STEM occupations, the importance of math, and the stereotyping image and beliefs for education, math, and women’s intellect. Research questions were:

- RQ1: Does providing information on “STEM occupations”, “gender equality in society” and “girls are good at math” increase the motivation of junior high school students to take STEM subjects?
- RQ2: Does providing these three information motivate parents to support their children to choose STEM subjects?

## Materials and methods

### Respondents

We conducted an online survey to investigate whether STEM-related information encouraged children to choose and participate in STEM, as well as their parents to support their children’s choice of STEM. In this study, we targeted first-year junior high school students, as they were unlikely to have settled on an interest in STEM. Their parents were also targeted, as their support was essential for children to choose science careers.

The authors contracted with NTT Research, Inc, a research company in Japan that collected the data using their data pool. The respondents agreed to the terms and conditions for handling personal information when they first registered with the company as a research monitor. The company sent an email to Japanese people who had a child in the first year of junior high school. We explained the purpose of this survey and privacy policy on the screen before they started to answer the questionnaire. Only parents who agreed to these conditions, and their children, could answer the questionnaire. We collected the data in child-parent pairs from 1,089 children (boys = 544, girls = 545) and their 1,089 parents (men = 534, women = 555). The combinations were son and father (n = 268), daughter and father (n = 266), son and mother (n = 276), and daughter and mother (n = 279). More than 40% of the parents graduated from university or more. The last school they graduated were junior high school (n = 23), high school (n = 293), undergraduate’s (n = 413), master’s (n = 51), doctor’s (n = 17), other’s (n = 2). All responses were considered valid. This study received approval from the Institutional Ethics Committee of the University of Tokyo (no. 19–419). The survey was conducted from March 24 to 26, 2020.

### Procedures

#### Experimental design

The online experiment had three stages: a pre-phase, information provision phase, and a post-phase. In the pre-phase, respondents replied to eight items (child) or five items (parent), and four quiz questions related to text paragraphs that we provided (child and parent). In the information provision phase, the respondents read the text. In the post-phase, the respondents replied to eight items (child) or five items (parent), and again answered the quiz questions (child and parent). The items and quiz questions in the pre- and post-phases were the same. The aim was to investigate whether their responses changed between the pre- and post-phases. The time required to complete all items and quizzes was about 10 to 15 minutes.

#### Questionnaire items

The questionnaire consisted of the following items.

- Q1. Motivation to choose STEM. They were asked “If you want to go to university, do you want to think positively about going on to mathematics, physics, engineering, or information science (science and technology)? For science and engineering majors, physics and chemistry are the most common subjects for science examinations.”
- Q2. Motivation to participate in STEM events. They were asked “Do you want to participate in STEM events for junior high school students and/or high school students?”
- Q3. Motivation to encourage their children to choose STEM. They were asked “When your junior high school student is considering university, do you want to actively support your child to go on to a science course in mathematics, physics, engineering, or information (science and technology)? For science and engineering majors, physics and chemistry are the most common subjects for science examinations.”

Q1 to Q3 were asked in the pre- and post-phases. Q1 and Q2 were only for children, and Q3 was only for parents. Their responses were rated on a five-point scale from agree (= 1) to disagree (= 5). For analysis, the scale assignments were reversed to indicate that higher scores indicated higher motivation.

The perceptions of gender equality, of three non-stereotypical images and beliefs, and of the perception of STEM occupations and learning were measured using six items for children (Q4–Q9) and four items for parents (Q4–Q7).

- Q4. SESRA-S (Scale of Egalitarian Sex Role Attitudes) score. Individual egalitarian attitudes toward gender roles were measured using the short form of SESRA-S, which was developed in the field of psychology. SESRA-S consists of 15 items (for example, “women at home and men at work”), each rated on a five-point scale from strongly agree (= 1) to strongly disagree (= 5). The individual level of attitudes toward gender equality were shown by the total score for 15 items (from 15 to 75) as the SESRA-S score [40, 41]. The higher the total score, the higher the gender equality. The reliability coefficient of the items was reported as 0.91 [40].
- Q5. Non-stereotypical view of education. We asked the respondents how much they agreed or disagreed with the statement “a university education is more important for men than women” at the pre- and post-phases.
- Q6. Non-stereotypical view of math. We asked the respondents how much they agreed or disagreed with the statement “women are not as good at mathematics as men by nature” at the pre- and post-phases.
- Q7. Non-stereotypical view of women’s intellect. We asked the respondents how much they agreed or disagreed with the statement “women should be intellectual” at the pre- and post-phases.

Q5 to Q7 were asked in the pre- and post-phases. Q4-Q7 were for both children and parents. Their responses were rated on a five-point scale from strongly agree (= 1) to strongly disagree (= 5). For analysis, the scale assignments of Q7 were reversed. The higher scores indicate less stereotyping.

- Q8. Perception about occupations. The question was “Do you think it is important to earn a salary for a continuing occupation, such as being a permanent employee?”
- Q9. Perception about the importance of learning math. The question “Do you think that learning mathematics will give you more job opportunities when you become an adult?” was asked in the pre- and post-phases.

Q8 and Q9 were asked in the pre- and post-phases for children. Their responses were rated on a five-point scale from agree (= 1) to disagree (= 5). For analysis, the scale assignments were reversed to indicate that higher scores indicated higher motivation.

#### Information

We prepared four types of information. Each consisted of short paragraphs of texts in Japanese with an illustration of a woman. The first information was on social media usage in Japan (referred to as “social media”, 413 words in Japanese). The second was on STEM occupations in Japan (referred to as “occupations”, 300 words). The third was entitled gender equal society in Japan (referred to as “society”, 448 words). The fourth was girls are good at math in Japan (referred to as “math”, 381 words). The three information (“occupations”, “society”, and “math”) mentioned gender equality and included some sentences focusing on girls, which encouraged them, but it was not included in the information on “social media” (Examples in English are shown in S1 Text, and in Japanese in S2 Text). Information on “social media” was used as the control information, and other three information were used as the treatment or experimental information.

Also, we prepared four quiz questions corresponding to each information to make sure that the respondents had read the information. As the limitation of online survey, we always have unconcentrated careless readers. The quizzes were not to test the respondents’ reading comprehension, but rather to confirm that they had read the information correctly. All the respondents were asked to answer the four quiz questions (either “yes” or “no”) regardless of which information they were given. The quizzes:

- Quiz question for “social media”. “The most used social media in Japan is Twitter.” (correct answer = “no”)
- Quiz question for “occupations”. “One of the areas of expertise that companies are looking for is mechanical engineering.” (correct answer = “yes”)
- Quiz question “society”. “In the Gender Gap Index (2019), which measures gender equality, Japan was in the top 100 out of 153 countries.” (correct answer = “no”)
- Quiz question “math”. “Female students in Japan perform lower in mathematics than boys.” (correct answer = “no”)

We provided four groups with combination of those information as shown in Table 1. The existing campaigns to encourage girls to choose STEM in Japan have conventionally provided girls with information of STEM occupations. But the information on gender equal society and girls’ math ability have not rarely provided to girls. We thus decided to investigate what additional information of “society” and “math” would be effective, on the basis that information “occupations” is always given. The first Treatment Group (occupations) read the information only on “occupations”. The second Treatment Group (occupations & society) read the information both on “occupations” and “society”. The third Treatment Group (occupations & math) read the information both on “occupations” and “math”. Control Group (social media) read the information on “social media”.

**Table 1:** Each group and combinations of information.

Group	Information			
	Social media	Occupations	Society	Math
Control Group (social media)	✓			
Treatment Group (occupations)		✓		
Treatment Group (occupations & society)		✓	✓	
Treatment Group (occupations & math)		✓		✓

#### Groups

A pair (one child and their parent) was randomly assigned to one of the four groups (Control Group (social media), Treatment Group (occupations), Treatment Group (occupations & society), Treatment Group (occupations & math)). A child and their parent were always assigned to the same group. The percentages of correct answers to each quiz question in pre-phase by children and parents are shown in Table 2.

**Table 2:** Correct answer (%) to each quiz questions in pre-phase.

	Social media	Occupations	Society	Math
Children	65.41	44.57	62.22	67.15
Parents	67.67	53.62	62.22	67.87

We regressed each demographic variable on the four groups (Control Group (social media) served as the baseline) to investigate whether the control and treatment groups were balanced. Age, gender of the children and parents, education of the parents, and major subjects at university (only respondents with an undergraduate degree or higher) of parents were well balanced among the four groups (Table 3).

**Table 3:** Profiles of the four groups of parents and children.

Variable	Explanation of variable	Raw means						Difference		
		Control Group (social media)	Treatment Group (occupations)	Treatment Group (occupations & society)	Treatment Group (occupations & math)			(Treatment Group (occupations))- (Control Group (social media))	(Treatment Group (occupations & society)- (Control Group (social media))	(Treatment Group (occupations & math))- (Control Group (social media))
		(1)	(2)	(3)	(4)			(5)	(6)	(7)
Age of Parents	Years of age	44.88 ± 5.91	45.49 ± 5.42	45.83 ± 5.45	45.60 ± 5.77	B		0.61	0.94	0.72
						95% Confidence Interval for (B)	Lower bound	-0.34	-0.01	-0.23
							Upper bound	1.56	1.90	1.66
						*p*		0.210	0.050	0.140
						R^2^ = 0.004				
Gender of Children	= 1 if Female, = 0 if Male	0.49	0.51	0.50	0.50	B		0.02	0.01	0.01
						95% Confidence Interval for (B)	Lower bound	-0.07	-0.07	-0.07
							Upper bound	0.10	0.10	0.10
						*p*		0.670	0.730	0.760
						R^2^ < 0.001				
Gender of Parents	= 1 if Female, = 0 if Male	0.51	0.51	0.51	0.51	B		0.00	0.00	0.00
						95% Confidence Interval for (B)	Lower bound	-0.09	-0.09	-0.08
							Upper bound	0.08	0.08	0.09
						*p*		0.930	0.930	0.970
						R^2^ < 0.001				
Education of Parents	= 1 if graduated from university or graduate university, = 0 otherwise	0.45	0.50	0.42	0.39	B		0.05	-0.03	-0.06
						95% Confidence Interval for (B)	Lower bound	-0.03	-0.11	-0.14
							Upper bound	0.13	0.06	0.03
						*p*		0.250	0.500	0.180
						R^2^ = 0.006				
Major course (only who graduated from university or graduate university) of Parents	= 1 if science, agriculture, engineering, or medical, = 0 otherwise	0.14	0.17	0.16	0.15	B		0.04	0.02	0.01
						95% Confidence Interval for (B)	Lower bound	-0.02	-0.04	-0.05
							Upper bound	0.10	0.09	0.07
						*p*		0.210	0.440	0.680
						R^2^ = 0.002				

This table presents statistics and estimated differences between Control Group (social media: those who are provided information on “social media”, Treatment Group (occupations: those who are provided information on “occupations”), Treatment Group (occupations & society: those who are provided information on “occupations & society”) and Treatment Group (occupations & math: those who are provided information on “occupations” and “math”). Column (1)-(4) presents means of age and rates of gender, education and major course; column (5) presents estimated differences between Treatment Group (occupations) and Control Group (social media); column (6) presents estimated differences between Treatment Group (occupations & society) and Control Group (social media), and column (7) presents estimated differences between Treatment Group (occupations & math) and Control Group (social media). Estimates in columns (5), (6) and (7) result from OLS regressions. Significance at the one- and five-percent levels is indicated by ** and *, respectively.

We regressed each item (Q1–Q9) in the pre-phase for the four groups (Control Group (social media) served as the baseline) to investigate whether the four groups were balanced (Table 4).

**Table 4:** Motivations and attitudes in children and parents in the pre-phase.

		Raw means						Difference		
		Control Group (social media)	Treatment Group (occupations)	Treatment Group (occupation & society)	Treatment Group (occupation & math)			(Treatment Group (occupations))- (Control Group (social media))	(Treatment Group (occupations & society))- (Control Group (social media))	(Treatment Group (occupations & math))- (Control Group (social media))
		(1)	(2)	(3)	(4)			(5)	(6)	(7)
*Children*										
Q1	Motivation to choose STEM	3.07	2.99	2.96	2.94	B		-0.07	-0.11	-0.13
						95% Confidence Interval for (B)	Lower bound	-0.24	-0.27	-0.30
							Upper bound	0.09	0.06	0.03
						*p*		0.372	0.198	0.113
						R^2^ =	0.003			
Q2	Motivation to participate in STEM events	3.06	3.06	3.03	3.01	B		0.00	-0.03	-0.05
						95% Confidence Interval for (B)	Lower bound	-0.18	-0.21	-0.23
							Upper bound	0.17	0.14	0.13
						*p*		0.979	0.704	0.581
						R^2^ =	0.000			
Q4	SESRA-S score	48.95	49.75	48.66	50.29	B		0.45	-0.49	1.32
						95% Confidence Interval for (B)	Lower bound	-0.83	-1.78	0.04
							Upper bound	1.74	0.80	2.61
						*p*		0.489	0.453	0.043*
						R^2^ =	0.008			
Q5	Non-stereotypical view of education	3.37	3.39	3.28	3.45	B		0.03	-0.08	0.08
						95% Confidence Interval for (B)	Lower bound	-0.12	-0.23	-0.07
							Upper bound	0.18	0.07	0.23
						*p*		0.723	0.274	0.271
						R^2^ =	0.005			
Q6	Non-stereotypical view of math skills	3.45	3.38	3.38	3.56	B		-0.07	-0.07	0.12
						95% Confidence Interval for (B)	Lower bound	-0.22	-0.22	-0.04
							Upper bound	0.09	0.09	0.27
						*p*		0.389	0.399	0.135
						R^2^ =	0.007			
Q7	Non-stereotypical view of women’s intellect	3.30	3.30	3.24	3.31	B		0.00	-0.06	0.01
						95% Confidence Interval for (B)	Lower bound	-0.14	-0.21	-0.13
							Upper bound	0.14	0.08	0.16
						*p*		1.000	0.387	0.855
						R^2^ =	0.001			
Q8	Occupations	3.97	3.94	3.99	4.01	B		-0.03	0.02	0.04
						95% Confidence Interval for (B)	Lower bound	-0.18	-0.13	-0.11
							Upper bound	0.13	0.18	0.20
						*p*		0.723	0.773	0.568
						R^2^ =	0.001			
Q9	Learning math	3.45	3.46	3.47	3.49	B		0.01	0.02	0.04
						95% Confidence Interval for (B)	Lower bound	-0.15	-0.14	-0.12
							Upper bound	0.16	0.18	0.20
						*p*		0.948	0.812	0.621
						R^2^ =	0.000			
*Parents*										
Q3	Motivation to encourage their children to choose STEM	3.64	3.50	3.53	3.54	B		-0.14	-0.11	-0.09
						95% Confidence Interval for (B)	Lower bound	-0.31	-0.28	-0.26
							Upper bound	0.03	0.07	0.08
						*p*		0.117	0.236	0.309
						R^2^ =	0.002			
Q4	SESRA-S score	50.37	50.90	49.92	52.23	B		0.51	-0.42	2.04
						95% Confidence Interval for (B)	Lower bound	-0.94	-1.88	0.58
							Upper bound	1.97	1.05	3.49
						*p*		0.491	0.576	0.006**
						R^2^ =	0.012			
Q5	Non-stereotypical view of education	3.55	3.51	3.44	3.59	B		-0.04	-0.11	0.04
						95% Confidence Interval for (B)	Lower bound	-0.21	-0.28	-0.13
							Upper bound	0.13	0.06	0.21
						*p*		0.659	0.211	0.614
						R^2^ =	0.003			
Q6	Non-stereotypical view of math skills	3.55	3.61	3.54	3.73	B		0.06	-0.01	0.18
						95% Confidence Interval for (B)	Lower bound	-0.10	-0.17	0.02
							Upper bound	0.23	0.16	0.34
						*p*		0.444	0.923	0.030*
						R^2^ =	0.006			
Q7	Non-stereotypical view of women’s intellect	3.45	3.41	3.39	3.42	B		-0.04	-0.06	-0.03
						95% Confidence Interval for (B)	Lower bound	-0.19	-0.21	-0.18
							Upper bound	0.11	0.09	0.12
						*p*		0.620	0.448	0.708
						R^2^ =	0.001			

This table presents statistics and estimated differences between Control Group (social media: those who are provided Information on “social media”), Treatment Group (occupations: those who are provided information on “occupations”), Treatment Group (occupations & society: those who are provided information on “occupations” and “society”) and Treatment Group (occupations & math: those who are provided information on “occupations” and “math”). Column (1)-(4) presents means; column (5) presents estimated differences between Treatment Group (occupations) and Control Group (social media); column (6) presents estimated differences between Treatment Group (occupations & society), and Control Group (social media), and column (7) presents estimated differences between Treatment Group (occupations & math) and Control Group (social media). Estimates in columns (5), (6) and (7) result from OLS regressions. Significance at the one- and five-percent levels is indicated by **and *, respectively.

For children, the motivation to choose STEM (Q1) and participate in STEM events (Q2) was balanced between the control and each treatment group. A significant difference was found in the SESRA-S score (Q4) in the Treatment Group (occupations & math) (*p* = 0.043), suggesting that the egalitarian attitude toward gender roles in Treatment Group (occupations & math) was higher than that in Control Group (social media). Thus, while the information was provided randomly, their egalitarian attitude was not balanced.

For parents, a significant difference was found in the SESRA-S score (Q4) between the Control Group (social media) and Treatment Group (occupations & math) (*p* = 0.006), meaning that the egalitarian attitude towards gender roles in Treatment Group (occupations & math) was higher than that in Control Group (social media). A significant difference was also found in the non-stereotypical view of math skills (Q6) between Control Group (social media) and Treatment Group (occupations & math) (*p* = 0.030), meaning that the Treatment Group (occupations & math) disagreed that women were not as good at mathematics as men by nature more than Control Group (social media). These results suggest that parents were balanced between the control and treatment groups across observable characteristics, but not balanced in their attitude of gender stereotypes. Since the differences may be related to the outcomes, we controlled for the SESRA-S score in the pre-phase for estimating the effect of providing STEM-related information.

### Analysis

First, we regressed the scores for each questionnaire item in the post-phase minus the pre-phase on the four groups (Control Group (social media) served as the baseline) controlling for respondents’ profiles (parents: age, gender, education and major subject at university; children: gender). We also controlled for baseline value (pre-phase) of SESRA-S score and response to each quiz question, looking at whether respondents correctly answered the question (corresponding to the text information) after providing information and baseline values (pre-phase) of each outcome.

Second, to examine whether the effects of providing gender equality information differ by gender, we added intersection terms between each group and gender to the model described in the first analysis, and estimated it. Third, to check whether the effectiveness of the information provision depends on the understanding, we add intersection terms between each group and responses to each quiz question to the model described in the first analysis, and estimated it. All analyses were conducted using IBM SPSS Statistics 25 software.

## Results

### Quizzes

The percentages of correct answers for each quiz in the post phase by information (Table 5), and by group (Table 6) was shown.

**Table 5:** Correct response to quiz question in post phase.

	Social media	Occupations	Society	Math
Children	71.43	52.17	64.07	77.98
Parents	75.94	56.16	64.44	80.51

**Table 6:** Correct response to quiz question in post phase by group.

	Control Group (social media)	Treatment Group (occupations)	Treatment Group (occupations & society)	Treatment Group (occupations & math)
Children	71.43	52.17	22.96	39.35
Parents	75.94	56.16	26.67	41.88

### First analysis

The scores for each questionnaire item in the post-phase minus the pre-phase were regressed on the four groups and covariates (Table 7).

**Table 7:** First analysis of changes in the motivation, perception, or others in children and parents between the post- and pre-phases.

			Children								Parents				
			Q1	Q2	Q4	Q5	Q6	Q7	Q8	Q9	Q3	Q4	Q5	Q6	Q7
			Motivation to choose STEM	Motivation to participate in STEM events	SESRA-S score	Non-stereotypical view of education	Non-stereotypical view of math skills	Non-stereotypical view of women’s intellect	Occupations	Learning math	Motivation to encourage their children to choose STEM	SESRA-S score	Non-stereotypical view of education	Non-stereotypical view of math skills	Non-stereotypical view of women’s intellect
Treatment Group (occupations)	B		0.13	0.05	0.59	0.04	-0.07	0.00	0.06	0.10	0.10	0.50	0.03	-0.05	-0.02
	95% Confidence Interval for (B)	Lower bound	0.01	-0.05	-0.06	-0.08	-0.19	-0.10	-0.04	-0.02	-0.02	-0.28	-0.10	-0.18	-0.15
		Upper bound	0.24	0.16	1.24	0.15	0.05	0.11	0.17	0.22	0.21	1.28	0.17	0.08	0.11
	*p*		0.029*	0.306	0.076	0.518	0.244	0.965	0.246	0.088	0.093	0.211	0.614	0.463	0.770
Treatment Group (occupations & society)	B		0.17	0.04	0.93	-0.01	-0.05	-0.02	0.06	0.11	0.02	0.56	0.09	0.01	-0.12
	95% Confidence Interval for (B)	Lower bound	0.04	-0.07	0.24	-0.13	-0.17	-0.13	-0.06	-0.01	-0.10	-0.27	-0.05	-0.13	-0.26
		Upper bound	0.29	0.15	1.62	0.10	0.07	0.10	0.17	0.23	0.14	1.39	0.23	0.15	0.01
	*p*		0.008**	0.491	0.008**	0.813	0.440	0.792	0.314	0.085	0.718	0.186	0.228	0.868	0.075
Treatment Group (occupations & math)	B		0.23	0.16	0.91	-0.03	0.07	0.06	0.07	0.18	0.13	1.07	0.06	0.16	-0.06
	95% Confidence Interval for (B)	Lower bound	0.11	0.05	0.25	-0.15	-0.05	-0.05	-0.05	0.06	0.01	0.26	-0.08	0.02	-0.19
		Upper bound	0.35	0.26	1.57	0.08	0.19	0.17	0.18	0.30	0.25	1.87	0.19	0.30	0.07
	*p*		0.000**	0.003**	0.007**	0.575	0.254	0.273	0.242	0.003**	0.032*	0.009**	0.412	0.021*	0.382
	R^2^		0.18	0.11	0.04	0.23	0.19	0.12	0.17	0.24	0.24	0.09	0.26	0.29	0.15

Results were from OLS regressions controlling for the participants’ profiles (parents: age, gender, education and major course at university, children: gender), SESRA-S score in pre phase, each outcome in pre phase and response to quizzes after providing information. Significance at the one- and five-percent levels is indicated by ** and *, respectively.

#### Change in motivations for STEM (Q1–Q3)

The coefficient of children’s motivation to choose STEM (Q1) was positively significant in Treatment Group (occupations) (*p* = 0.029), Treatment Group (occupations & society) (*p* = 0.008), and Treatment Group (occupations & math) (*p* < 0.001). This suggests that providing information on “occupations”, information on “society” and “occupations” together, or information on “math” and “society” together increased their motivation more than information on “social media”. The coefficient of children’s motivation to participate in STEM events (Q3) was positively significant in Treatment Group (occupations & math) (*p* < 0.014).

For parents, there was a positive significant difference in the motivation to encourage their children to choose STEM (Q3) in Treatment Group (occupations & math) (*p* = 0.032). These results demonstrate that providing information on “occupations” and “math” together increased both children’s and their parent’s motivation for STEM.

#### Change in stereotypical views and other factors (Q4–Q9)

For children, there was a positive significant difference in learning math (Q9) in Treatment Group (occupations & math) (*p* = 0.003). This suggests that providing information on “occupation” and “math” together, which is about there being no gender gap in mathematics, improved their attitudes to math. For the parents, positive significances were found in the coefficients of SESRA-S (Q4, *p* = 0.009) and the non-stereotypical views of math skills (Q6) in Treatment Group (occupations & math) (*p* = 0.021), suggesting that providing information on “occupation” and “math” together increased their egalitarian attitudes and decreased math stereotyping.

### Second analysis

The scores for each questionnaire item in the post-phase minus the pre-phase were regressed on the four groups, the interaction between the four groups and the gender of respondents, and covariates (S2 Table).

#### Change in motivations for STEM (Q1–Q3)

There was not a significant difference in the interaction between the four groups and gender in motivation to choose STEM (Q1) and motivation to participate in STEM event (Q2) in children, and motivation to encourage their children to choose STEM (Q3) in parents. This suggests that providing gender equality information does not discourage STEM choices for boys.

#### Change in stereotypical views and other factors (Q4–Q9)

For children, there was a positive significant difference in learning math (Q9) in the interaction between Treatment Group (occupations & society) and sex (girls) (*p* = 0.046), meaning that the information on “occupations” and “society” together improved girls’ attitudes to math. For parents, there were not a significant difference in the interactions in Q4 to Q7.

### Third analysis

To investigate relationships between groups and responses to the quizzes, we regressed the score of each questionnaire item in the post-phase minus the pre-phase for the four groups, as well as responses to quizzes in the post-phase and interactions between each group and responses to quiz questions (Table 8).

**Table 8:** Third analysis of change in the motivation, perception, or others in children and parents between the post- and pre-phases.

			Children								Parents				
			Q1	Q2	Q4	Q5	Q6	Q7	Q8	Q9	Q3	Q4	Q5	Q6	Q7
			Motivation to choose STEM	Motivation to participate in STEM events	SESRA-S score	Non-stereotypical view of education	Non-stereotypical view of math skills	Non-stereotypical view of women’s intellect	Occupations	Learning math	Motivation to encourage their children to choose STEM	SESRA-S score	Non-stereotypical view of education	Non-stereotypical view of math skills	Non-stereotypical view of women’s intellect
Treatment Group (occupations)	B		-0.05	0.01	0.86	-0.01	0.03	-0.05	0.04	-0.08	-0.06	1.09	0.16	0.10	-0.15
	95% Confidence Interval for (B)	Lower bound	-0.24	-0.16	-0.22	-0.20	-0.16	-0.22	-0.14	-0.28	-0.26	-0.30	-0.07	-0.13	-0.37
		Upper bound	0.14	0.18	1.94	0.18	0.22	0.13	0.22	0.11	0.14	2.48	0.40	0.34	0.08
	*p*		0.602	0.895	0.117	0.924	0.754	0.610	0.694	0.384	0.558	0.124	0.168	0.386	0.201
Treatment Group (occupations & society)	B		0.04	0.02	0.84	-0.09	-0.09	0.00	0.08	-0.05	-0.12	0.47	0.15	0.08	-0.28
	95% Confidence Interval for (B)	Lower bound	-0.14	-0.14	-0.16	-0.27	-0.27	-0.16	-0.09	-0.23	-0.31	-0.82	-0.07	-0.14	-0.49
		Upper bound	0.22	0.18	1.85	0.08	0.09	0.17	0.24	0.12	0.07	1.77	0.37	0.30	-0.07
	*p*		0.656	0.827	0.099	0.290	0.339	0.973	0.379	0.546	0.206	0.472	0.169	0.475	0.009 **
Treatment Group (occupations & math)	B		0.10	0.16	1.10	-0.07	0.02	0.06	0.06	0.01	-0.07	0.82	0.15	0.16	-0.24
	95% Confidence Interval for (B)	Lower bound	-0.08	-0.01	0.06	-0.25	-0.16	-0.11	-0.11	-0.17	-0.27	-0.51	-0.08	-0.06	-0.46
		Upper bound	0.29	0.33	2.13	0.11	0.21	0.23	0.24	0.19	0.12	2.15	0.37	0.39	-0.02
	*p*		0.270	0.058	0.038 *	0.457	0.803	0.498	0.472	0.915	0.479	0.227	0.196	0.154	0.029 *
Correct answer	B		-0.01	0.08	1.02	-0.04	0.02	0.05	0.09	-0.05	-0.02	0.67	0.15	0.18	-0.06
	95% Confidence Interval for (B)	Lower bound	-0.19	-0.08	0.00	-0.22	-0.17	-0.12	-0.08	-0.23	-0.21	-0.62	-0.07	-0.04	-0.27
		Upper bound	0.17	0.24	2.04	0.14	0.20	0.22	0.26	0.13	0.17	1.96	0.37	0.40	0.15
	*p*		0.904	0.336	0.049 *	0.655	0.866	0.551	0.310	0.577	0.860	0.310	0.175	0.109	0.552
Treatment Group (occupations)*Correct answer	B		0.28	0.07	-0.48	0.06	-0.19	0.09	0.05	0.28	0.22	-1.04	-0.20	-0.25	0.16
	95% Confidence Interval for (B)	Lower bound	0.04	-0.14	-1.84	-0.17	-0.44	-0.13	-0.18	0.04	-0.03	-2.73	-0.48	-0.54	-0.11
		Upper bound	0.52	0.29	0.88	0.30	0.05	0.31	0.28	0.52	0.46	0.65	0.09	0.04	0.44
	*p*		0.022 *	0.511	0.487	0.599	0.117	0.429	0.647	0.023 *	0.088	0.227	0.178	0.092	0.241
Treatment Group (occupations & society) *Correct answer	B		0.19	0.04	0.62	0.20	0.16	-0.09	-0.07	0.27	0.18	0.40	-0.06	-0.11	0.25
	95% Confidence Interval for (B)	Lower bound	-0.08	-0.20	-0.86	-0.06	-0.11	-0.34	-0.32	0.01	-0.08	-1.39	-0.36	-0.41	-0.04
		Upper bound	0.45	0.28	2.11	0.46	0.42	0.15	0.17	0.53	0.45	2.18	0.25	0.20	0.54
	*p*		0.166	0.730	0.412	0.124	0.250	0.451	0.558	0.046 *	0.167	0.664	0.719	0.488	0.093
Treatment Group (occupations & math) *Correct answer	B		0.18	-0.02	-0.37	0.03	0.11	0.00	0.01	0.26	0.32	0.63	-0.13	0.06	0.29
	95% Confidence Interval for (B)	Lower bound	-0.06	-0.25	-1.75	-0.20	-0.13	-0.23	-0.23	0.02	0.07	-1.06	-0.42	-0.23	0.01
		Upper bound	0.43	0.20	1.00	0.27	0.36	0.23	0.24	0.51	0.57	2.32	0.15	0.35	0.56
	*p*		0.138	0.827	0.593	0.783	0.363	0.994	0.964	0.036 *	0.012 *	0.465	0.365	0.693	0.040 *
	R^2^	0.18	0.11	0.04	0.23	0.20	0.12	0.17	0.24	0.25	0.10	0.26	0.29	0.15

Notes: Results were from OLS regressions controlling for the participants’ profiles (parents: age, gender, education and major course at university, children: gender), baseline value (pre phase) of SESRA-S score and each outcome. Significance at the five percent levels is indicated by *.

#### Change in motivations for STEM (Q1–Q3)

Coefficient of children’s motivation to choose STEM (Q1) were positively significant in the interaction between Treatment Group (occupations) and the correct quiz answer (*p* = 0.022), suggesting that children’s motivations in the Treatment groups (occupations) increased especially in those who correctly answered the quiz question on “occupation” compared with children in the same group who answered the quiz incorrectly. There were no significant differences in the interaction between Treatment Group (occupations & society) and the correct quiz answer (*p* = 0.166) and between Treatment Group (occupations & math) and the correct quiz answer (*p* = 0.138), but the coefficients were positive values.

The coefficient of parents’ motivation to encourage their children to choose STEM (Q3) was positively significant in the interaction between Treatment Group (occupations & math) and the correct answer (*p* = 0.012), indicating that the parents in Treatment Group (occupations & math) who answered quiz question on “math” correctly increased their motivation compared with those who incorrectly answered the same quiz question.

#### Change in stereotypical views and other factors (Q4–Q9)

For children’s learning math (Q9), a positive significance was found in Treatment Group (occupations) and the correct quiz answer (*p* = 0.023); Treatment Group (occupations & society) and the correct quiz answer (*p* = 0.046); Treatment Group (occupations & math) and the correct quiz answer (*p* = 0.036) meaning that children who answered the quiz question corresponding to the information previously provided to them correctly increased their desire to learn math compared with those who answered quiz question incorrectly.

For parents, regarding the non-stereotypical view of women’s intellect (Q7), a positive significance in the interaction between Treatment group (occupations & math) were found (*p* = 0.040). This suggests that the parents in Treatment Group (occupations & society) who answered the quiz question on “occupation” and “math” decreased their stereotypical view compared with those who answered the quizzes incorrectly.

## Findings

- Children had a higher motivation to choose STEM after they read information about gender equal society (“society”) or that girls are good at math (“math”) in addition to STEM occupations (“occupations”).
- Parents increased their motivation to encourage their children to choose STEM after they read the information about girls being good at math in addition to STEM occupations. This effect was stronger in those who read the information then correctly answered the quiz question.

## Discussion

We investigated whether providing three types of gender quality information motivates children to choose and participate in STEM as well as their parents’ motivation to support their children to choose STEM subjects. We found that information about girls being good at math (information on “math”) in addition to STEM occupations (information on “occupations”) increased both children’s (Q1) and their parents’ motivation for STEM (Q3). This suggests that providing this information is an effective way to momentarily at least motivate both children and their parents. Furthermore, since there were no gender differences in the effects of providing this information, it is unlikely that there is a negative effect of discouraging boys from intending to major in STEM. However, it remains unclear if they will really transform their behavior, learn STEM and support their children to learn STEM over time.

The percentage of correct answers to quiz questions (a)-(c) before information was read in the pre-phase was relatively low. Especially, correct answer percentage was lowest for quiz on “occupations” (children, 44.57%; parents, 53.62%, Table 2), which asked whether mechanical engineering was an area of expertise sought by companies. This indicates that about half of the children and their parents had not known and recognized that people who have studied mechanical engineering are in high demand. This knowledge gap could be one reason that girls less often choose mechanical engineering at university. We need to be proactive in communication to students that mechanical engineering is in demand in society, for example, through STEM events for junior high school students.

On the other hand, the percentage of correct answers in the pre-phase were the highest (66–68%) for quiz question on “math” (children, 67.15%; parents, 67.87%, Table 2), which asked whether Japanese female students perform lower than boys in mathematics. The percentage of correct answers was around 70%, In other words, 30% believed that girls are not as good as boys at mathematics. This suggests that there are still many children and parents who have a mathematical stereotypical view in Japan. Efforts are also needed at policy level to reduce a mathematical stereotypical view. For the question about children learning math (Q9), it is noteworthy that information on “occupation”, “society” and “math” increased the children’s motivation to learn math if they read the information correctly (Table 8). When reading information on STEM, children may realize that mathematics is important to them and stimulate their motivation.

One interesting finding was that information on “society” did not change parents’ motivation to encourage their children to choose STEM (Q3). Information on “society” directly explained the current gender unequal situation in Japan. This might make the parents face the reality and feel that it would be difficult to change the situation, which might help to maintain their gender-unequal recognition.

This online survey had limitations. First, there were many respondents who did not read the information correctly. A study showed that many people respond to online questionnaires without fully reading the instructions [42]. It is likely that some of the respondents in our study only glanced at the questionnaire and responded without reading the information. We prepared quiz questions to identify those respondents who understood the information correctly. As a result, the percentage who understood the information correctly was around 50% for all the quizzes (Table 5). For the other 50%, those who answered incorrectly, we still cannot identify if they did not read the information or they read the information but could not understand it. One suggestion here is that just providing information is not enough to have people read and understand it. A process to confirm whether they read the information would improve data reliability and their understanding.

Second, we need to be careful that the effect confirmed was very temporary (about 10–15 minutes). Our results showed that children’s motivation toward STEM increased by the provision of information, but this does not mean that their behaviors change. It remains unclear whether this effect can last over the long term or until the children choose their courses at high school.

Third, we found that the effect of providing information on “occupations” and “math” was stronger for parents in several cases. However, the parents in Treatment Group (occupation & math) had higher egalitarian attitudes than other groups (Table 4). This means that although we controlled for the differences in egalitarian attitudes among groups, we cannot exclude the possibility that the respondents with higher egalitarian attitudes are likely to change their motivation to support their children to choose STEM.

Fourth, we asked parents to participate in the survey with their children but to respond separately when answering. However, we could not confirm whether the respondents complied with this request. As a future experiment, collecting responses from parents and children separately might be better. The effect of providing information was not the same for children and their parents, which suggests that it is likely that the respondents did follow our rule about answering separately.

At last, we need to mention that most of the questionnaire items except SESRA-S score was assessed using only one single item. Further study of the statistical validity of these items is needed.

In summary, our findings show that providing gender equality information, especially information on math stereotypes, and STEM occupations, is effective in increasing both children’s and their parents’ motivation for STEM. Information on STEM occupations have been frequently provided to children at schools and STEM-related events. Our results propose that actively providing information that Japanese girls do mathematics very well in addition to the existing information providing may empower children to study STEM. Also, it would be beneficial to develop policies to motivate children towards STEM through the provision of information, as the provision of information itself is relatively low-cost activity. Further study is necessary to investigate whether the effect of providing information lasts to motivate children and parents over the long term.

## Supporting information

S1 Text Scenarios in English. (PDF)

S2 Text Scenarios in Japanese. (PDF)

S1 Table Full model of first analysis. (PDF)

S2 Table Second analysis of change in the motivation, perception, or others in children and parents between the post- and pre-phases. (PDF)

S3 Table Full model of second analysis. (PDF)

S4 Table Full model of third analysis. (PDF)

S1 File Raw data. (XLSX)

## References

[1] Bargh JA, Chen M, Burrows L. Automaticity of social behavior: direct effects of trait construct and stereotype-activation on action. Journal of Personality and Social Psychology. 1996; 71(2): 230–244. doi: 10.1037//0022-3514.71.2.230 8765481

[2] Bargh JA, Chartrand TL. Studying the Mind in the Middle: A Practical Guide to Priming and Automaticity Research. In: Reis H, Judd C, editors. Handbook of Research Methods in Social Psychology. New York: Cambridge University Press; 2000. pp. 253–285.

[3] Ramsey LR, Betz DE, Sekaquaptewa D. The effects of an academic environment intervention on science identification among women in STEM. Social Psychology of Education. 2013; 16(3): 377–397. doi: 10.1007/s11218-013-9218-6

[4] Lin-Siegler X, Ahn JN, Chen J, Fang FFA, Luna-Lucero M. Even Einstein struggled: Effects of learning about great scientists’ struggles on high school students’ motivation to learn science. Journal of Educational Psychology. 2016; 108(3): 314–328. doi: 10.1037/edu0000092

[5] Moss-Racusin CA, Pietri ES, Hennes EP, Dovidio JF, Brescoll VL, Roussos G, et al. Reducing STEM gender bias with VIDS (video interventions for diversity in STEM). Journal of Experimental Psychology: Applied. 2018: 24(2); 236–260. doi: 10.1037/xap0000144 29733620

[6] Starr CR, Anderson BR, Green KA. “I’m a Computer Scientist!”: Virtual Reality Experience Influences Stereotype Threat and STEM Motivation Among Undergraduate Women. Journal of Science Education and Technology. 2019; 28(5): 493–507. doi: 10.1007/s10956-019-09781-z

[7] Finger C, Solga H, Ehlert M, Rusconi A. Gender differences in the choice of field of study and the relevance of income information. Insights from a field experiment. Research in Social Stratification and Mobility. 2019; 100457. doi: 10.1016/j.rssm.2019.100457

[8] Jasko K, Dukala K, Szastok M. Focusing on gender similarities increases female students’ motivation to participate in STEM. Journal of Applied Social Psychology. 2019; 49(8): 473–487. doi: 10.1111/jasp.12598

[9] Ministry of Economy, Trade and Industry. Rikōkeijinzai ikusei ni kakaru genjōbunseki dēta nado. (in Japanese). [Data analysis of the current situation regarding the development of human resources in the field of engineering and technology]. [Internet]. 2016. Available from: https://www.meti.go.jp/policy/innovation_corp/entaku/pdf/data.pdf

[10] Gender Equity Bureau Cabinet Office. The act on promotion of women’s participation and advancement in the workplace [Internet]. n.d. Available from: http://www.gender.go.jp/english_contents/about_danjo/lbp/pdf/promotion_of_woman.pdf

[11] Ministry of Health, Labour and Welfare. Josei katsuyakusuishin no torikumi kōjireishu. (in Japanese). [Collection of best practices for promoting women’s activities] [Internet]. 2018. Available from: https://www.mhlw.go.jp/file/06-Seisakujouhou-11900000-Koyoukintoujidoukateikyoku/0000197011.pdf

[12] Ikkatai Y, Minamizaki A, Kano K, Inoue A, McKay E, Yokoyama, HM. Gender-biased public perception of STEM fields, focusing on the influence of egalitarian attitudes toward gender roles. Journal of Science Communication. 2020; 19(1): A08. doi: 10.22323/2.19010208

[13] Benesse Corporation. Shinro sentaku ni kansuru furukaeri chōsa (in Japanese) [A retrospective study of career choices] [Internet]. 2005. Available from: https://berd.benesse.jp/berd/center/open/report/shinrosentaku/2005/pdf/shinrosentaku01.pdf

[14] Libertas Consulting Co., Ltd. Zenkoku gakuryoku, gakushū jōkyo chōsa no kekka o mochiita rikani taisuru iyoku, kanshin nado ga chūgakko dankai de teikasuru yoin ni kansuru chōsa kenkyū. (in Japanese). [A Study on Factors of Decline in Motivation and Interest in Science at Junior High School Using the Results of the National Assessment of Educational Progress and Learning] [Internet]. 2014. Available from: https://www.mext.go.jp/component/a_menu/education/micro_detail/__icsFiles/afieldfile/2015/08/24/1361058_02.pdf

[15] Harada Y, Sakamoto K, Suzuki M. When and why have lower secondary school students disliked science learning?: a basic study based on expectancy-value theory. Journal of Research in Science Education. 2018; 58(3): 319–330. doi: 10.11639/sjst.17028

[16] National Institute for Educational Policy Research of Japan. TIMSS2015 sansū sūgaku kyōiku/rika kyōiku no kokusaihikaku. (in Japanese) [TIMSS 2015 International comparison of math and mathematics education/science education]. Akashi Shoten; 2017.

[17] Ministry of Education, Culture, Sports, Science and Technology. TIMSS2015 oyobi PISA2015 no kokusai kekka ni tsuite. (in Japanese). [International results of TIMSS 2015 and PISA 2015] [Internet]. 2018. Available from https://www.koho2.mext.go.jp/208/voice/208_03.html

[18] OECD. Programme for international student assessment (PISA) results from PISA 2018 [Internet]. 2019. Available from: https://www.oecd.org/pisa/publications/PISA2018_CN_JPN_Japanese.pdf

[19] OECD. Education at a Glance 2019: OECD Indicators [Internet]. Paris: OECD Publishing; 2019. Available from: 10.1787/f8d7880d-en. p.23

[20] Smith J. 14 high-paying jobs for people who love math [Internet]. Business insider; 2016 Jun 15. Available from: https://www.businessinsider.com/high-paying-jobs-for-people-who-love-math-2016-6#-12

[21] U.S. Bureau of labor statistics. Data table for the overview of May 2019 occupational employment and wages [Internet]. 2020 March 31. Available from: https://www.bls.gov/oes/2019/may/featured_data.htm#stem3

[22] Beede DN, Julian TA, Langdon D, McKittrick G, Khan B, Doms ME. Women in STEM: A gender gap to innovation. Economics and Statistics Administration Issue Brief, 2011; 04–11: 1–11. https://ssrn.com/abstract=1964782

[23] Ministry of Health, Labour and Welfare. Basic Survey on Wage Structure. (in Japanese). [Internet]. 2020. Available from: https://www.e-stat.go.jp/stat-search/files?page=1&toukei=00450091&tstat=000001011429

[24] World Economic Forum. Global Gender Gap Report 2020 [Internet]. 2019. Available from: http://www3.weforum.org/docs/WEF_GGGR_2020.pdf

[25] Ministry of Education, Culture, Sports, Science and Technology. Reiwa gannendo gakkō kihon chōsa sokuhochi no kōhyo ni tsuite. (in Japanese). [Announcement of the Preliminary Report of the school basic survey 2019] [Internet]. 2019. Available from: https://www.mext.go.jp/component/b_menu/other/__icsFiles/afieldfile/2019/08/08/1419592_1.pdf

[26] Japan Broadcasting Corporation. Dai jikkai nihonjin no ishiki chōsa kekka no gaiyō. (in Japanese) [Results of the 10th Annual Japanese Awareness Survey (2018)] [Internet]. n.d. Available from: https://www.nhk.or.jp/bunken/research/yoron/pdf/20190107_1.pdf

[27] Gakkō Kihon Chōsa. Daigaku, Daigakuin. (in Japanese). [University, Graduate university] [Internet]. 2019. Available from: https://www.e-stat.go.jp/stat-search/files?page=1&layout=datalist&toukei=00400001&tstat=000001011528&cycle=0&tclass1=000001135783&tclass2=000001135810&tclass3=000001135811&tclass4=000001135813

[28] Holloway S, Yamamoto Y, Suzuki S. Exploring the Gender Gap: Women Speak Out about Working and raising children in contemporary Japan [Internet]. Child research net; 2005 January 1. Available from: https://www.childresearch.net/papers/parenting/2005_03.html

[29] Amano M. Women in higher education. Higher Education. 1997; 34(2): 215–235. doi: 10.1023/A:1003051803994

[30] Tenenbaum HR, Leaper C. Parent-child conversations about science: The socialization of gender inequities?. Developmental Psychology, 2003; 39(1): 34–47. doi: 10.1037//0012-1649.39.1.34 12518807

[31] Bleeker MM, Jacobs JE. Achievement in math and science: Do mothers’ beliefs matter 12 years later?. Journal of Educational Psychology. 2004; 96(1): 97–109. doi: 10.1037/0022-0663.96.1.97

[32] Gender Equity Bureau Cabinet Office. Ricō-challenge [Internet]. n.d. Available from: http://www.gender.go.jp/c-challenge/

[33] Japan Science and Technology Agency. Joshichūkōseino rikeishinro sentaku shiem puroguramu. (in Japanese). [Program to support girls in choosing a career in science] [Internet]. n.d. Available from: https://www.jst.go.jp/cpse/jyoshi/

[34] Kodansha Ltd. Rikejo [Internet]. n.d. Available from: https://www.rikejo.jp/

[35] Google. Kompyūtā saiensuo mijikani–Mind the Gap puroguramu. (in Japanese). [Computer Science Made Accessible—Mind the Gap Program] [Internet]. 2016 May 24. Available from: https://japan.googleblog.com/2016/05/mindthegap.html

[36] Gakkō Kihon Chōsa. Daigaku. (in Japanese). [University] [Internet]. 1989. Available from: https://www.e-stat.go.jp/stat-search/files?page=1&layout=datalist&toukei=00400001&tstat=000001011528&cycle=0&tclass1=000001071057&tclass2=000001071105&tclass3=000001071113&tclass4=000001071115

[37] Kahn S, Ginther D. Women and STEM. NBER Working Paper. 2017; 23525: 1–42. doi: 10.3386/w23525

[38] Spencer SJ, Steele CM, Quinn DM. Stereotype threat and women’s math performance. Journal of Experimental Social Psychology. 1999; 35(1): 4–28. doi: 10.1006/jesp.1998.1373

[39] OECD. PISA 2018 Results Combined Executive Summaries Volume I, II, & IIII [Internet]. Paris: OECD Publishing; 2019. Available from: https://www.oecd.org/pisa/Combined_Executive_Summaries_PISA_2018.pdf

[40] Suzuki A. Construction of a short-form of the scale of egalitarian sex role attitudes (SESRA-S). The Japanese Journal of Psychology. 1994; 65(1): 34–41. doi: 10.4992/jjpsy.65.34 8022127

[41] Ui M. Jendā Seiyakuwari (in Japanese). [Gender and gender roles] In: Hori H, Yamamoto M, editors. Shinri Sokutei Syakudoshu II. (in Japanese) [Psychometric Scale Collection II]. Tokyo, Japan: SAIENSU-SHA Co. Ltd; 2001. pp. 137–172.

[42] Miura A, Kobayashi T. Mechanical Japanese: Survey satisficing of online panels in Japan. Japanese Journal of Social Psychology. 2015; 31(1): 1–12. doi: 10.14966/jssp.31.1_1

