# Supplementary material for: Effect of providing gender equality information on students’ motivations to choose STEM
Source: PLoS One. 2021 Jun 23;16(6):e0252710. doi: 10.1371/journal.pone.0252710 (PMC8221466; doi:10.1371/journal.pone.0252710)
Supplement: S1 Text — (PDF) [file pone.0252710.s001.pdf]

S1 Text. Scenarios in English.

Scenario (social media)

We constructed the texts in information on “social media usage” based on the information provided by Ministry of Internal Affairs and communication [1], which is used as a control information.

> **Social media usage**. Since 2000, the amount of time spent watching TV in Japan has been slowly declining. On the other hand, the time spent on the Internet has increased significantly. This trend is especially common in the 20s. How about social media usage? Social media includes a variety of services such as Facebook, Twitter, LINE and other social networking services, other online chats, blogs, information and review sharing sites, bulletin boards, mailing lists, and online games. In Japan, LINE has the highest rate of usage, with about 60% of the users including those who use it even a little. As an overall trend, the percentage of users who answered that they rarely send out information or speak out, and only read other people's posts and remarks, was higher than those who actively write on any of the social media platforms. In the U.S, the U.K. and Germany, more people use Facebook than in Japan, and a higher percentage of people write frequently.

Scenario (occupations)

We constructed the texts in information on “STEM occupations” based on the information provided by Ministry of Economy, Trade and Industry [2].

> **STEM occupations**. With the current focus on AI (artificial intelligence), many people with high IT-related expertise are in demand, and many people from mathematics and physics departments, as well as information-related departments, are hired at related companies to develop AI. In addition to this, Japan's strong manufacturing industry requires a large number of people with strong expertise in mechanical engineering, power and electrical equipment. These STEM jobs are known to be stable and pay well. However, the percentage of women in Japan who choose to study science and technology is low, which is one of the reasons why there are so few women in stable jobs. Companies and the government are working to encourage and support women to study STEM and find STEM-related employment.

Scenario (gender equal society)

We reconstructed the texts in information on “gender equal society” based on the information of Global Gender Gap Index [3] and the current situation of Japanese society.

> **Gender equal society**. Japan has been slow to raise awareness of gender equality, and men are still the overwhelming majority at the top of politicians, companies, and schools. Japan is not a society that is easy for women to work in. The Gender Gap Index, which ranks each country's gender equality based on the number of politicians and gender differences in economic participation, is ranked 121st out of 153 countries (2019), which is very low, and is often pointed out by the international community as lagging behind in reform. Once you reach adulthood, regardless of your gender, the first step to independence is to work to make a living for yourself instead of having someone else provide for you. Working and earning a salary gives you the freedom to decide things at your own will. In today's society, it is necessary to create an environment in which both women and men can work comfortably, and at the same time, to have at least a certain number of women on the decision-making board of an organization. It is also considered desirable in today's society for women and women to continue working even after marriage, and for both women and men to handle domestic work and child rearing equally. Regardless your gender, it is your right to think and choose your way of living and your role in society.

Scenario (girls’ math)

We constructed the texts in information on “girls’ math” based on TIMSS2015 [4, 5].

> **Girls’ math**. Have you ever thought that boys are better suited to math? No, no, it's not like that. Girls are really good at math! In particular, Japanese girls' math skills are among the best in the world, and on the international test for eighth grade students (TIMSS 2015), Japanese girls scored just as well as boys, and this score was higher than boys in U.S.. If we translate the TIMSS scores into a familiar deviation score, Japanese girls are 3 points higher than boys, which means that the deviation is 0.3 points higher and there is little difference between boys and girls. Japanese girls are also 69 points higher than American boys, which means they have a 6.9 higher deviation score. Japanese students’ scores were good, ranking 5th out of 39 countries for both boys and girls. It is believed that maths performance depends on an individual difference, not gender, and that it is heavily

influenced by the country and environment.

References

1. Ministry of internal affairs and communication. White paper information and communications in Japan [Internet]. 2018. Available from: https://www.soumu.go.jp/johotsusintokei/whitepaper/eng/WP2018/2018-index.html
2. Ministry of Economy, Trade and Industry. Ricōkei jinzai ikusei ni kakaru genjōbunseki deta no seiri. (in Japanese) [Organize data for analysis of the current status of human resources development for science and engineering] [Internet]. 2016. Available from: https://www.meti.go.jp/policy/innovation_corp/entaku/pdf/160506_entaku8_shiryo01.pdf
3. World Economic Forum. Global Gender Gap Report 2020 [Internet]. 2019. Available from: http://www3.weforum.org/docs/WEF_GGGR_2020.pdf
4. Ministry of Education, Culture, Sports, Science and Technology. TIMSS2015 oyobi PISA2015 no kokusai kekka ni tsuite. (in Japanese). [International results of TIMSS 2015 and PISA 2015] [Internet]. 2018. Available from https://www.koho2.mext.go.jp/208/voice/208_03.html
5. National Institute for Educational Policy Research of Japan. TIMSS2015 sansū sūgaku kyōiku/rika kyōiku no kokusaihikaku. (in Japanese) [TIMSS 2015 International comparison of math and mathematics education/science education]. Akashi Shoten; 2017.
