# Supplementary material for: Effect of providing gender equality information on students’ motivations to choose STEM
Source: PLoS One. 2021 Jun 23;16(6):e0252710. doi: 10.1371/journal.pone.0252710 (PMC8221466; doi:10.1371/journal.pone.0252710)
Supplement: S2 Text — (PDF) [file pone.0252710.s002.pdf]

S2 Text. Scenarios in Japanese.

Scenario (social media)

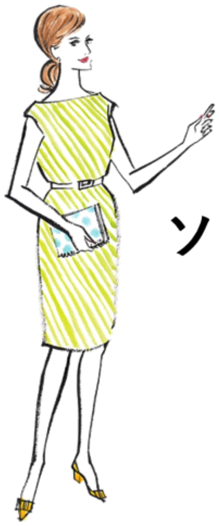

ソーシャルメディアの利用状況

２０００年以降、日本ではテレビの視聴時間は緩やかに減少しています。その一方で、インターネットの利用時間は大幅に増加しています。この傾向は特に２０代でよく見られます。

ソーシャルメディアの利用状況はどうでしょうか。ソーシャルメディアには、Facebook、Twitter、LINE、その他のSNS、その他のオンラインチャット、ブログ、情報・レビュー共有サイト、掲示板、メーリングリスト、オンラインゲームなど様々なサービスが含まれます。日本ではLINEの利用率が最も高く、少しでも利用している人を含めた利用者の割合はおよそ６０％です。全体的な傾向として、いずれのソーシャルメディアでも「ほとんど情報発信や発言せず、他人の書き込みや発言等の閲覧しか行わない」と回答する利用者の割合が、書き込みなどを行う利用者よりも多いことが分かりました。アメリカ、イギリス、ドイツではFacebookの利用者が日本と比較して多く、さらに頻繁に書き込みをしている割合が高いことが分かりました。

Scenario (occupations)

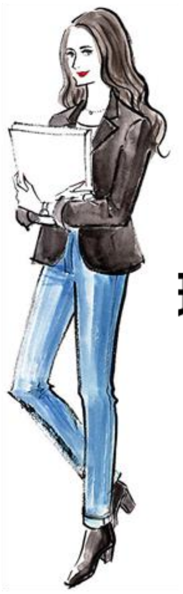

理工系就職のニーズ

企業において必要な専門分野は幅広くあります。ＡＩ（人工知能）が注目をされている現在、ＩＴ関係の専門性の高い人材は多く求められており、情報系の学科のみならず、数学科や物理学科などからも多くの人材がＡＩ開発のために関連企業に就職をしています。またそれにも増して製造業に強い日本は、機械工学や、電力、電気機器の専門性の高い人材を多く必要としています。

こうした理工系の就職は、安定的で給与もよいことが知られています。しかし日本の女性は、理工系に進学をする割合が少なく、安定的な職業に女性が少ない要因にもなっています。

企業や政府は、女性の理工系への進学、就職に期待をし、応援をする活動をしています。

Scenario (gender equal society)

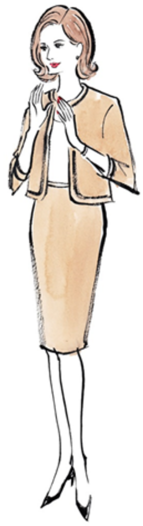

平等社会

日本は、男女平等の意識改革が遅れており、政治家や企業、学校のトップはいまだに男性が圧倒的多数です。また、女性にとって働きやすい社会とは言い難い状況です。ジェンダーギャップ指数と呼ばれる、政治家の人数や経済参加の男女差から、それぞれの国の男女の平等度をランキングする指標では、１５３か国中１２１位（２０１９年）と大変低い状態で、国際社会から改革が遅れていると指摘を受けることも少なくありません。

成人すれば性別に関わらず、他の人に養ってもらうのではなく、自分で生活するために働くことが自立の第一歩です。働いて給与を得ることは、自分の意思で物事を決定する自由を得ることにつながります。現代社会では、女性も男性も働きやすい環境を整えると同時に、組織の意思決定をする役員などに、女性が一定以上いることが求められています。また、現代では結婚しても男女ともに仕事を続け、家事・子育てを男女共に平等に行う社会が望ましいと考えられています。

性別に関わらず、自分の生き方や、社会での役割を考え、選ぶのは、皆さんの権利です。

Scenario (girls' math)

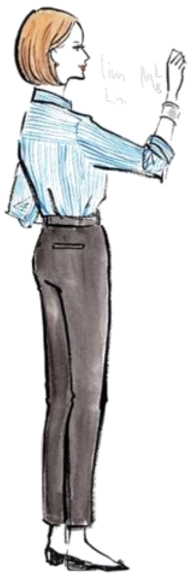

女子は数学が得意

数学は男子の方が向いていると思ったことはありませんか。いえいえ、そんなことはありません。女子は数学が得意なのです！特に日本の女子生徒の数学力は、世界的に見ても高く、中学２年生の生徒が受ける国際試験（ＴＩＭＳＳ２０１５）では、日本の女子生徒の点数は男子と変わりなく、さらにこの点数は、アメリカの男子よりも高かったのです。

ＴＩＭＳＳの点数を皆さんにとってなじみのある偏差値に置き換えると、日本の女子生徒の点数は男子生徒よりも３点高いですが、このことは偏差値が０.３高いことを意味し、男女差はほとんど見られません。また日本の女子生徒はアメリカの男子よりも６９点高いですが、このことは、偏差値が６.９高いことを意味しています。日本の成績は、男女合わせて３９か国中５位の好成績でした。

数学の成績は、性別ではなく、個人の違いであること、また国や環境による影響が大きいと考えられています。
