# Supplementary material for: Effect of providing gender equality information on students’ motivations to choose STEM
Source: PLoS One. 2021 Jun 23;16(6):e0252710. doi: 10.1371/journal.pone.0252710 (PMC8221466; doi:10.1371/journal.pone.0252710)
Supplement: S1 Table — (PDF) [file pone.0252710.s003.pdf]

S1 Table. Full model of first analysis.

Results of children:

Q1: Motivation to choose STEM

	Unstandardized Coefficients		Standardized Coefficients	t	Sig.	95% Confidence Interval for (B)	
	B	Std. Error	β			Lower bound	Upper bound
(Constant)	0.63	0.16		3.93	0.000	0.31	0.94
Q4_children_pre (Children's SESRA-S score in the pre-phase)	0.01	0.00	0.06	2.01	0.045	0.00	0.01
Q1_children_pre (Children's motivation to choose STEM in the pre-phase)	-0.31	0.02	-0.40	-14.18	0.000	-0.35	-0.26
Gender_children (= girl)	-0.07	0.04	-0.05	-1.67	0.095	-0.15	0.01
Treatment group (occupations & math)	0.23	0.06	0.14	3.84	0.000	0.11	0.35
Treatment group (occupations & society)	0.17	0.06	0.10	2.67	0.008	0.04	0.29
Treatment group (occupation)	0.13	0.06	0.08	2.19	0.029	0.01	0.24
Quizzes_children_post (= Correct answer to quiz corresponding to the information in post-phase)	0.16	0.04	0.11	3.58	0.000	0.07	0.25

Q2: Motivation to participate in STEM events

	Unstandardized Coefficients		Standardized Coefficients	t	Sig.	95% Confidence Interval for (B)	
	B	Std. Error	β			Lower bound	Upper bound
(Constant)	0.28	0.14		2.03	0.042	0.01	0.55
Q4_children_pre (Children's SESRA-S score in the pre-phase)	0.00	0.00	0.06	2.00	0.046	0.00	0.01
Q2_children_pre (Children's motivation to participate in "STEM-events" in the pre-phase)	-0.20	0.02	-0.32	-10.94	0.000	-0.23	-0.16
Gender_children (= girl)	-0.02	0.04	-0.01	-0.47	0.636	-0.09	0.06
Treatment group (occupations & math)	0.16	0.05	0.11	2.93	0.003	0.05	0.26
Treatment group (occupations & society)	0.04	0.06	0.03	0.69	0.491	-0.07	0.15
Treatment group (occupation)	0.05	0.05	0.04	1.02	0.306	-0.05	0.16
Quizzes_children_post (= Correct answer to quiz corresponding to the information in post-phase)	0.10	0.04	0.08	2.57	0.010	0.02	0.18

Q4: SESRA-S score

	Unstandardized Coefficients		Standardized Coefficients	t	Sig.	95% Confidence Interval for (B)	
	B	Std. Error	β			Lower bound	Upper bound
(Constant)	3.25	0.79		4.10	0.000	1.70	4.81
Q4_children_pre (Children's SESRA-S score in the pre-phase)	-0.08	0.02	-0.16	-5.21	0.000	-0.11	-0.05
Gender_children (= girl)	0.10	0.23	0.01	0.44	0.664	-0.35	0.56
Treatment group (occupations & math)	0.91	0.34	0.10	2.69	0.007	0.25	1.57
Treatment group (occupations & society)	0.93	0.35	0.10	2.65	0.008	0.24	1.62
Treatment group (occupation)	0.59	0.33	0.07	1.77	0.076	-0.06	1.24
Quizzes_children_post (= Correct answer to quiz corresponding to the information in post-phase)	0.90	0.25	0.12	3.61	0.000	0.41	1.39

Q5: Non-stereotypical view of education

	Unstandardized Coefficients		Standardized Coefficients	t	Sig.	95% Confidence Interval for (B)	
	B	Std. Error	β			Lower bound	Upper bound
(Constant)	0.20	0.14		1.48	0.140	-0.07	0.47
Q4_children_pre (Children's SESRA-S score in the pre-phase)	0.03	0.00	0.31	9.10	0.000	0.02	0.04
Q5_children_pre (Children's stereotypical view of education in the pre-phase)	-0.52	0.03	-0.60	-17.74	0.000	-0.57	-0.46
Gender_children (= girl)	0.02	0.04	0.01	0.41	0.680	-0.06	0.10
Treatment group (occupations & math)	-0.03	0.06	-0.02	-0.56	0.575	-0.15	0.08
Treatment group (occupations & society)	-0.01	0.06	-0.01	-0.24	0.813	-0.13	0.10
Treatment group (occupation)	0.04	0.06	0.02	0.65	0.518	-0.08	0.15
Quizzes_children_post (= Correct answer to quiz corresponding to the information in post-phase)	0.03	0.04	0.02	0.66	0.511	-0.06	0.11

Q6: Non-stereotypical view of math skills

	Unstandardized Coefficients		Standardized Coefficients	t	Sig.	95% Confidence Interval for (B)	
	B	Std. Error	β			Lower bound	Upper bound
(Constant)	0.14	0.14		0.97	0.331	-0.14	0.42
Q4_children_pre (Children's SESRA-S score in the pre-phase)	0.03	0.00	0.28	8.49	0.000	0.02	0.03
Q6_children_pre (Children's stereotypical view of math skills in the pre-phase)	-0.44	0.03	-0.53	-16.01	0.000	-0.49	-0.39
Gender_children (= girl)	-0.06	0.04	-0.04	-1.34	0.182	-0.14	0.03
Treatment group (occupations & math)	0.07	0.06	0.04	1.14	0.254	-0.05	0.19
Treatment group (occupations & society)	-0.05	0.06	-0.03	-0.77	0.440	-0.17	0.07
Treatment group (occupation)	-0.07	0.06	-0.04	-1.16	0.244	-0.19	0.05
Quizzes_children_post (= Correct answer to quiz corresponding to the information in post-phase)	0.02	0.04	0.01	0.47	0.640	-0.07	0.11

Q7: Non-stereotypical view of women’s intellect

	Unstandardized Coefficients		Standardized Coefficients	t	Sig.	95% Confidence Interval for (B)	
	B	Std. Error	β			Lower bound	Upper bound
(Constant)	0.49	0.14		3.40	0.001	0.21	0.77
Q4_children_pre (Children's SESRA-S score in the pre-phase)	0.01	0.00	0.09	2.95	0.003	0.00	0.01
Q7_children_pre (Children's stereotypical view of women's intellect in the pre phase)	-0.26	0.02	-0.33	-11.58	0.000	-0.30	-0.22
Gender_children (= girl)	0.01	0.04	0.01	0.18	0.854	-0.07	0.08
Treatment group (occupations & math)	0.06	0.06	0.04	1.10	0.273	-0.05	0.17
Treatment group (occupations & society)	-0.02	0.06	-0.01	-0.26	0.792	-0.13	0.10
Treatment group (occupation)	0.00	0.05	0.00	0.04	0.965	-0.10	0.11
Quizzes_children_post (= Correct answer to quiz corresponding to the information in post-phase)	0.06	0.04	0.04	1.41	0.159	-0.02	0.14

Q8: Occupations

	Unstandardized Coefficients		Standardized Coefficients	t	Sig.	95% Confidence Interval for (B)	
	B	Std. Error	β			Lower bound	Upper bound
(Constant)	0.47	0.15		3.25	0.001	0.19	0.76
Q4_children_pre (Children's SESRA-S score in the pre-phase)	0.01	0.00	0.14	4.95	0.000	0.01	0.02
Q8_children_pre (Children's response to the occupations in the pre-phase)	-0.32	0.02	-0.42	-14.60	0.000	-0.36	-0.28
Gender_children (= girl)	-0.02	0.04	-0.02	-0.56	0.573	-0.10	0.05
Treatment group (occupations & math)	0.07	0.06	0.04	1.17	0.242	-0.05	0.18
Treatment group (occupations & society)	0.06	0.06	0.04	1.01	0.314	-0.06	0.17
Treatment group (occupation)	0.06	0.06	0.04	1.16	0.246	-0.04	0.17
Quizzes_children_post (= Correct answer to quiz corresponding to the information in post-phase)	0.09	0.04	0.06	2.15	0.032	0.01	0.17

Q9: Learning math

	Unstandardized Coefficients		Standardized Coefficients	t	Sig.	95% Confidence Interval for (B)	
	B	Std. Error	β			Lower bound	Upper bound
(Constant)	0.37	0.15		2.42	0.016	0.07	0.67
Q4_children_pre (Children's SESRA-S score in the pre-phase)	0.02	0.00	0.18	6.57	0.000	0.01	0.02
Q9_children_pre (Children's response to learning math in the pre-phase)	-0.39	0.02	-0.47	-17.41	0.000	-0.43	-0.35
Gender_children (= girl)	-0.05	0.04	-0.03	-1.15	0.250	-0.13	0.03
Treatment group (occupations & math)	0.18	0.06	0.10	2.98	0.003	0.06	0.30
Treatment group (occupations & society)	0.11	0.06	0.06	1.72	0.085	-0.01	0.23
Treatment group (occupation)	0.10	0.06	0.06	1.71	0.088	-0.02	0.22
Quizzes_children_post (= Correct answer to quiz corresponding to the information in post-phase)	0.16	0.04	0.10	3.50	0.000	0.07	0.24

Results of parents:

Q3: Motivation to encourage their children to choose STEM

	Unstandardized Coefficients		Standardized Coefficients	t	Sig.	95% Confidence Interval for (B)	
	B	Std. Error	β			Lower bound	Upper bound
(Constant)	-1.70	0.24		-7.19	0.000	-2.16	-1.24
age_p (Age of parents)	0.01	0.00	0.04	1.49	0.136	0.00	0.01
Q4_parents_pre (Parental SESRA-S score in the pre-phase)	0.00	0.00	0.05	1.83	0.067	0.00	0.01
Q3_parents_pre (Parental motivation to encourage their children to choose STEM in the pre-phase)	0.36	0.02	0.49	17.82	0.000	0.32	0.40
Treatment group (occupations & math)	0.13	0.06	0.07	2.14	0.032	0.01	0.25
Treatment group (occupations & society)	0.02	0.06	0.01	0.36	0.718	-0.10	0.14
Treatment group (occupation)	0.10	0.06	0.06	1.68	0.093	-0.02	0.21
Gender_parent (= women)	0.07	0.05	0.05	1.48	0.140	-0.02	0.16
major (= Science/agriculture/engineering/medicine)	0.15	0.07	0.07	2.33	0.020	0.02	0.28
education (= Those who graduated from university or graduate university)	0.05	0.05	0.03	1.07	0.286	-0.04	0.15
Quizzes_parent_post (= Correct answer to quiz corresponding to the information in post-phase)	0.18	0.04	0.11	3.98	0.000	0.09	0.26

Q4: SESRA-S score

	Unstandardized Coefficients		Standardized Coefficients	t	Sig.	95% Confidence Interval for (B)	
	B	Std. Error	β			Lower bound	Upper bound
(Constant)	2.57	1.55		1.66	0.098	-0.48	5.62
age_p (Age of parents)	0.06	0.03	0.07	2.31	0.021	0.01	0.12
Q4_parents_pre (Parental SESRA-S score in the pre-phase)	-0.15	0.02	-0.27	-9.12	0.000	-0.18	-0.12
Treatment group (occupations & math)	1.07	0.41	0.10	2.61	0.009	0.26	1.87
Treatment group (occupations & society)	0.56	0.42	0.05	1.32	0.186	-0.27	1.39
Treatment group (occupation)	0.50	0.40	0.05	1.25	0.211	-0.28	1.28
Gender_parent (= women)	1.17	0.32	0.12	3.63	0.000	0.54	1.81
major (= Science/agriculture/engineering/medicine)	-0.88	0.45	-0.07	-1.97	0.049	-1.76	0.00
education (= Those who graduated from university or graduate university)	1.35	0.33	0.14	4.07	0.000	0.70	2.00
Quizzes_parent_post (= Correct answer to quiz corresponding to the information in post-phase)	0.64	0.30	0.07	2.11	0.035	0.04	1.23

Q5: Non-stereotypical view of education

	Unstandardized Coefficients		Standardized Coefficients	t	Sig.	95% Confidence Interval for (B)	
	B	Std. Error	β			Lower bound	Upper bound
(Constant)	0.18	0.26		0.70	0.481	-0.33	0.70
age_p (Age of parents)	0.00	0.00	0.03	0.89	0.372	0.00	0.01
Q4_parents_pre (Parental SESRA-S score in the pre-phase)	0.03	0.00	0.25	7.72	0.000	0.02	0.03
Q5_parents_pre (Parental stereotypical view of education in the pre-phase)	-0.54	0.03	-0.61	-18.67	0.000	-0.60	-0.48
Treatment group (occupations & math)	0.06	0.07	0.03	0.82	0.412	-0.08	0.19
Treatment group (occupations & society)	0.09	0.07	0.04	1.20	0.228	-0.05	0.23
Treatment group (occupation)	0.03	0.07	0.02	0.50	0.614	-0.10	0.17
Gender_parent (= women)	0.07	0.05	0.04	1.33	0.182	-0.03	0.18
major (= Science/agriculture/engineering/medicine)	-0.12	0.08	-0.05	-1.53	0.127	-0.26	0.03
education (= Those who graduated from university or graduate university)	0.11	0.06	0.06	2.02	0.043	0.00	0.22
Quizzes_parent_post (= Correct answer to quiz corresponding to the information in post-phase)	0.04	0.05	0.02	0.88	0.381	-0.05	0.14

Q6: Non-stereotypical view of math skills

	Unstandardized Coefficients		Standardized Coefficients	t	Sig.	95% Confidence Interval for (B)	
	B	Std. Error	β			Lower bound	Upper bound
(Constant)	0.41	0.26		1.56	0.120	-0.11	0.93
age_p (Age of parents)	0.00	0.00	0.01	0.23	0.816	-0.01	0.01
Q4_parents_pre (Parental SESRA-S score in the pre-phase)	0.03	0.00	0.25	8.50	0.000	0.02	0.03
Q6_parents_pre (Parental stereotypical view of math skills in the pre-phase)	-0.57	0.03	-0.60	-20.46	0.000	-0.63	-0.52
Treatment group (occupations & math)	0.16	0.07	0.08	2.31	0.021	0.02	0.30
Treatment group (occupations & society)	0.01	0.07	0.01	0.17	0.868	-0.13	0.15
Treatment group (occupation)	-0.05	0.07	-0.02	-0.73	0.463	-0.18	0.08
Gender_parent (= women)	0.01	0.06	0.01	0.17	0.861	-0.10	0.12
major (= Science/agriculture/engineering/medicine)	-0.12	0.08	-0.05	-1.52	0.128	-0.27	0.03
education (= Those who graduated from university or graduate university)	0.17	0.06	0.09	2.99	0.003	0.06	0.28
Quizzes_parent_post (= Correct answer to quiz corresponding to the information in post-phase)	0.10	0.05	0.05	1.96	0.050	0.00	0.20

Q7: Non-stereotypical view of women’s intellect

	Unstandardized Coefficients		Standardized Coefficients	t	Sig.	95% Confidence Interval for (B)	
	B	Std. Error	β			Lower bound	Upper bound
(Constant)	0.86	0.26		3.35	0.001	0.36	1.36
age_p (Age of parents)	0.00	0.00	0.01	0.17	0.868	-0.01	0.01
Q4_parents_pre (Parental SESRA-S score in the pre-phase)	0.00	0.00	0.04	1.23	0.221	0.00	0.01
Q7_parents_pre (Parental stereotypical view of women's intellect in the pre phase)	-0.34	0.03	-0.38	-12.98	0.000	-0.39	-0.29
Treatment group (occupations & math)	-0.06	0.07	-0.03	-0.87	0.382	-0.19	0.07
Treatment group (occupations & society)	-0.12	0.07	-0.07	-1.78	0.075	-0.26	0.01
Treatment group (occupation)	-0.02	0.06	-0.01	-0.29	0.770	-0.15	0.11
Gender_parent (= women)	0.11	0.05	0.07	2.10	0.036	0.01	0.21
major (= Science/agriculture/engineering/medicine)	0.01	0.07	0.01	0.16	0.870	-0.13	0.15
education (= Those who graduated from university or graduate university)	0.07	0.05	0.04	1.28	0.201	-0.04	0.18
Quizzes_parent_post (= Correct answer to quiz corresponding to the information in post-phase)	0.12	0.05	0.07	2.44	0.015	0.02	0.22
