# Supplementary material for: Effect of providing gender equality information on students’ motivations to choose STEM
Source: PLoS One. 2021 Jun 23;16(6):e0252710. doi: 10.1371/journal.pone.0252710 (PMC8221466; doi:10.1371/journal.pone.0252710)
Supplement: S2 Table — (PDF) [file pone.0252710.s004.pdf]

S2 Table. Second analysis of change in the motivation, perception, or others in children and parents between the post- and pre-phases.

			Children								Parents				
			Q1	Q2	Q4	Q5	Q6	Q7	Q8	Q9	Q3	Q4	Q5	Q6	Q7
			Motivation to choose STEM	Motivation to participate in STEM events	SESRA-S score	Non-stereotypical view of education	Non-stereotypical view of math skills	Non-stereotypical view of women's intellect	Occupations	Learning math	Motivation to encourage their children to choose STEM	SESRA-S score	Non-stereotypical view of education	Non-stereotypical view of math skills	Non-stereotypical view of women's intellect
Treatment Group (occupations)	B		0.17	-0.01	0.87	0.04	-0.02	0.00	0.05	0.10	0.03	0.65	0.00	-0.03	-0.11
	95% Confidence Interval for (B)	Lower bound	0.01	-0.16	-0.05	-0.11	-0.18	-0.15	-0.11	-0.06	-0.13	-0.46	-0.19	-0.22	-0.29
		Upper bound	0.33	0.13	1.78	0.20	0.15	0.15	0.20	0.26	0.19	1.76	0.19	0.16	0.07
	p		0.042 *	0.868	0.063	0.579	0.839	0.966	0.534	0.225	0.709	0.249	0.997	0.741	0.215
Treatment Group (occupations & society)	B		0.25	-0.05	1.17	0.08	0.03	-0.09	0.07	-0.01	-0.02	0.55	0.04	0.00	-0.19
	95% Confidence Interval for (B)	Lower bound	0.08	-0.20	0.22	-0.09	-0.14	-0.24	-0.09	-0.18	-0.19	-0.61	-0.15	-0.20	-0.38
		Upper bound	0.42	0.10	2.11	0.24	0.19	0.07	0.23	0.16	0.15	1.70	0.24	0.20	-0.01
	p		0.003 **	0.489	0.016 *	0.366	0.767	0.263	0.396	0.909	0.807	0.351	0.659	0.999	0.043 *
Treatment Group (occupations & math)	B		0.30	0.07	1.49	0.04	0.15	0.10	0.07	0.17	0.09	0.62	0.03	0.06	-0.13
	95% Confidence Interval for (B)	Lower bound	0.13	-0.07	0.57	-0.12	-0.02	-0.05	-0.09	0.00	-0.08	-0.52	-0.16	-0.13	-0.32
		Upper bound	0.46	0.22	2.41	0.20	0.31	0.25	0.22	0.33	0.25	1.75	0.22	0.25	0.05
	p		0.000 **	0.327	0.001 **	0.609	0.079	0.206	0.385	0.044 *	0.301	0.286	0.739	0.553	0.156
Sex (girls/women)	B		0.027	-0.142	0.666	0.105	0.049	-0.013	-0.024	-0.112	-0.006	1.029	0.023	-0.037	-0.009
	95% Confidence Interval for (B)	Lower bound	-0.137	-0.289	-0.254	-0.054	-0.115	-0.164	-0.179	-0.275	-0.176	-0.134	-0.173	-0.235	-0.199
		Upper bound	0.191	0.005	1.585	0.264	0.213	0.138	0.131	0.051	0.165	2.192	0.219	0.161	0.180
	p		0.746	0.058	0.156	0.196	0.559	0.864	0.757	0.178	0.948	0.083	0.818	0.717	0.923
Treatment Group (occupations) * sex (girls/women)	B		-0.08	0.14	-0.57	-0.02	-0.11	0.01	0.03	0.00	0.13	-0.31	0.07	-0.04	0.19
	95% Confidence Interval for (B)	Lower bound	-0.31	-0.07	-1.86	-0.24	-0.34	-0.20	-0.19	-0.23	-0.09	-1.86	-0.20	-0.30	-0.06
		Upper bound	0.15	0.34	0.71	0.20	0.12	0.22	0.25	0.23	0.36	1.24	0.33	0.23	0.44
	p		0.481	0.196	0.383	0.869	0.364	0.919	0.777	0.986	0.252	0.693	0.622	0.780	0.146
Treatment Group (occupations & society) * sex (girls/women)	B		-0.17	0.19	-0.49	-0.18	-0.15	0.14	-0.02	0.23	0.09	0.01	0.08	0.02	0.14
	95% Confidence Interval for (B)	Lower bound	-0.40	-0.02	-1.79	-0.41	-0.38	-0.07	-0.24	0.00	-0.14	-1.55	-0.18	-0.25	-0.11
		Upper bound	0.06	0.39	0.80	0.04	0.08	0.36	0.20	0.46	0.31	1.57	0.35	0.29	0.39
	p		0.144	0.078	0.457	0.112	0.204	0.182	0.867	0.046 *	0.465	0.993	0.540	0.881	0.282
Treatment Group (occupations & math) * sex (girls/women)	B		-0.13	0.17	-1.18	-0.15	-0.16	-0.07	0.00	0.02	0.08	0.87	0.05	0.20	0.15
	95% Confidence Interval for (B)	Lower bound	-0.36	-0.03	-2.46	-0.37	-0.39	-0.28	-0.22	-0.21	-0.15	-0.68	-0.21	-0.07	-0.11
		Upper bound	0.09	0.38	0.11	0.07	0.07	0.14	0.21	0.25	0.31	2.42	0.31	0.46	0.40
	p		0.252	0.099	0.073	0.184	0.177	0.494	0.972	0.841	0.487	0.270	0.725	0.140	0.255
	R^2		0.18	0.12	0.04	0.23	0.20	0.12	0.17	0.24	0.24	0.09	0.26	0.29	0.15

Results were from ordinal regressions controlling for the participants' profiles (parents: age, gender, education and major course at university, children: gender), SESRA-S score in pre phase, each outcome in pre phase and response to quizzes after providing information outcome. Significance at the one- and five-percent levels is indicated by **and *, respectively.
