# Supplementary material for: Effect of providing gender equality information on students’ motivations to choose STEM
Source: PLoS One. 2021 Jun 23;16(6):e0252710. doi: 10.1371/journal.pone.0252710 (PMC8221466; doi:10.1371/journal.pone.0252710)
Supplement: S3 Table — (PDF) [file pone.0252710.s005.pdf]

S3 Table. Full model of second analysis.

Results of children:

Q1: Motivation to choose STEM

	Unstandardized Coefficients		Standardized Coefficients	t	Sig.	95% Confidence Interval for (B)	
	B	Std. Error	β			Lower bound	Upper bound
(Constant)	0.58	0.16		3.51	0.00	0.25	0.90
Gender_children (= girl)	0.03	0.08	0.02	0.32	0.75	-0.14	0.19
Treatment group (occupations & math)	0.30	0.08	0.17	3.57	0.00	0.13	0.46
Treatment group (occupations & society)	0.25	0.09	0.15	2.94	0.00	0.08	0.42
Treatment group (occupation)	0.17	0.08	0.10	2.04	0.04	0.01	0.33
Quizzes_children_post (= Correct answer to quiz corresponding to the information in post-phase)	0.16	0.04	0.11	3.57	0.00	0.07	0.25
Q4_children_pre (Children's SESRA-S score in the pre-phase)	0.01	0.00	0.06	2.00	0.05	0.00	0.01
Q1_children_pre (Children's motivation to choose STEM in the pre-phase)	-0.30	0.02	-0.40	-14.15	0.00	-0.35	-0.26
Girl * Treatment Group (occupations & math)	-0.13	0.12	-0.06	-1.15	0.25	-0.36	0.09
Girl * Treatment Group (occupations & society)	-0.17	0.12	-0.08	-1.46	0.14	-0.40	0.06
Girl * Treatment Group (occupations)	-0.08	0.12	-0.04	-0.71	0.48	-0.31	0.15

Q2: Motivation to participate in STEM events

	Unstandardized Coefficients		Standardized Coefficients	t	Sig.	95% Confidence Interval for (B)	
	B	Std. Error	β			Lower bound	Upper bound
(Constant)	0.34	0.14		2.41	0.02	0.06	0.62
Gender_children (= girl)	-0.14	0.08	-0.11	-1.89	0.06	-0.29	0.01
Treatment group (occupations & math)	0.07	0.07	0.05	0.98	0.33	-0.07	0.22
Treatment group (occupations & society)	-0.05	0.08	-0.04	-0.69	0.49	-0.20	0.10
Treatment group (occupation)	-0.01	0.07	-0.01	-0.17	0.87	-0.16	0.13
Quizzes_children_post (= Correct answer to quiz corresponding to the information in post-phase)	0.10	0.04	0.08	2.56	0.01	0.02	0.18
Q4_children_pre (Children's SESRA-S score in the pre-phase)	0.00	0.00	0.06	1.97	0.05	0.00	0.01
Q2_children_pre (Children's motivation to participate in "STEM-events" in the pre-phase)	-0.20	0.02	-0.32	-10.93	0.00	-0.23	-0.16
Girl * Treatment Group (occupations & math)	0.17	0.10	0.09	1.65	0.10	-0.03	0.38
Girl * Treatment Group (occupations & society)	0.19	0.11	0.10	1.76	0.08	-0.02	0.39
Girl * Treatment Group (occupations)	0.14	0.10	0.07	1.29	0.20	-0.07	0.34

Q4: SESRA-S score

	Unstandardized Coefficients		Standardized Coefficients	t	Sig.	95% Confidence Interval for (B)	
	B	Std. Error	β			Lower bound	Upper bound
(Constant)	2.93	0.82		3.55	0.00	1.31	4.54
Gender_children (= girl)	0.67	0.47	0.09	1.42	0.16	-0.25	1.59
Treatment group (occupations & math)	1.49	0.47	0.17	3.18	0.00	0.57	2.41
Treatment group (occupations & society)	1.17	0.48	0.13	2.42	0.02	0.22	2.11
Treatment group (occupation)	0.87	0.47	0.10	1.86	0.06	-0.05	1.78
Quizzes_children_post (= Correct answer to quiz corresponding to the information in post-phase)	0.89	0.25	0.12	3.57	0.00	0.40	1.39
Q4_children_pre (Children's SESRA-S score in the pre-phase)	-0.08	0.02	-0.16	-5.12	0.00	-0.11	-0.05
Girl * Treatment Group (occupations & math)	-1.18	0.66	-0.10	-1.79	0.07	-2.46	0.11
Girl * Treatment Group (occupations & society)	-0.49	0.66	-0.04	-0.74	0.46	-1.79	0.80
Girl * Treatment Group (occupations)	-0.57	0.66	-0.05	-0.87	0.38	-1.86	0.71

Q5: Non-stereotypical view of education

	Unstandardized Coefficients		Standardized Coefficients	t	Sig.	95% Confidence Interval for (B)	
	B	Std. Error	β			Lower bound	Upper bound
(Constant)	0.16	0.14		1.15	0.25	-0.12	0.44
Gender_children (= girl)	0.10	0.08	0.07	1.29	0.20	-0.05	0.26
Treatment group (occupations & math)	0.04	0.08	0.02	0.51	0.61	-0.12	0.20
Treatment group (occupations & society)	0.08	0.08	0.04	0.90	0.37	-0.09	0.24
Treatment group (occupation)	0.04	0.08	0.03	0.55	0.58	-0.11	0.20
Quizzes_children_post (= Correct answer to quiz corresponding to the information in post-phase)	0.03	0.04	0.02	0.63	0.53	-0.06	0.11
Q4_children_pre (Children's SESRA-S score in the pre-phase)	0.03	0.00	0.31	9.05	0.00	0.02	0.04
Q5_children_pre (Children's stereotypical view of education in the pre-phase)	-0.51	0.03	-0.60	-17.69	0.00	-0.57	-0.46
Girl * Treatment Group (occupations & math)	-0.15	0.11	-0.07	-1.33	0.18	-0.37	0.07
Girl * Treatment Group (occupations & society)	-0.18	0.11	-0.08	-1.59	0.11	-0.41	0.04
Girl * Treatment Group (occupations)	-0.02	0.11	-0.01	-0.17	0.87	-0.24	0.20

Q6: Non-stereotypical view of math skills

	Unstandardized Coefficients		Standardized Coefficients	t	Sig.	95% Confidence Interval for (B)	
	B	Std. Error	β			Lower bound	Upper bound
(Constant)	0.08	0.15		0.57	0.57	-0.21	0.37
Gender_children (= girl)	0.05	0.08	0.03	0.58	0.56	-0.12	0.21
Treatment group (occupations & math)	0.15	0.08	0.08	1.76	0.08	-0.02	0.31
Treatment group (occupations & society)	0.03	0.09	0.01	0.30	0.77	-0.14	0.19
Treatment group (occupation)	-0.02	0.08	-0.01	-0.20	0.84	-0.18	0.15
Quizzes_children_post (= Correct answer to quiz corresponding to the information in post-phase)	0.02	0.04	0.01	0.46	0.64	-0.07	0.11
Q4_children_pre (Children's SESRA-S score in the pre-phase)	0.03	0.00	0.28	8.47	0.00	0.02	0.03
Q6_children_pre (Children's stereotypical view of math skills in the pre-phase)	-0.44	0.03	-0.53	-15.96	0.00	-0.49	-0.39
Girl * Treatment Group (occupations & math)	-0.16	0.12	-0.07	-1.35	0.18	-0.39	0.07
Girl * Treatment Group (occupations & society)	-0.15	0.12	-0.07	-1.27	0.20	-0.38	0.08
Girl * Treatment Group (occupations)	-0.11	0.12	-0.05	-0.91	0.36	-0.34	0.12

Q7: Non-stereotypical view of women's intellect

	Unstandardized Coefficients		Standardized Coefficients	t	Sig.	95% Confidence Interval for (B)	
	B	Std. Error	β			Lower bound	Upper bound
(Constant)	0.49	0.15		3.30	0.00	0.20	0.78
Gender_children (= girl)	-0.01	0.08	-0.01	-0.17	0.86	-0.16	0.14
Treatment group (occupations & math)	0.10	0.08	0.06	1.27	0.21	-0.05	0.25
Treatment group (occupations & society)	-0.09	0.08	-0.06	-1.12	0.26	-0.24	0.07
Treatment group (occupation)	0.00	0.08	0.00	-0.04	0.97	-0.15	0.15
Quizzes_children_post (= Correct answer to quiz corresponding to the information in post-phase)	0.06	0.04	0.04	1.36	0.17	-0.02	0.14
Q4_children_pre (Children's SESRA-S score in the pre-phase)	0.01	0.00	0.09	3.05	0.00	0.00	0.01
Q7_children_pre (Children's stereotypical view of women's intellect in the pre phase)	-0.26	0.02	-0.33	-11.57	0.00	-0.30	-0.22
Girl * Treatment Group (occupations & math)	-0.07	0.11	-0.04	-0.68	0.49	-0.28	0.14
Girl * Treatment Group (occupations & society)	0.14	0.11	0.07	1.33	0.18	-0.07	0.36
Girl * Treatment Group (occupations)	0.01	0.11	0.01	0.10	0.92	-0.20	0.22

Q8: Occupations

	Unstandardized Coefficients		Standardized Coefficients	t	Sig.	95% Confidence Interval for (B)	
	B	Std. Error	β			Lower bound	Upper bound
(Constant)	0.48	0.15		3.16	0.00	0.18	0.77
Gender_children (= girl)	-0.02	0.08	-0.02	-0.31	0.76	-0.18	0.13
Treatment group (occupations & math)	0.07	0.08	0.04	0.87	0.38	-0.09	0.22
Treatment group (occupations & society)	0.07	0.08	0.04	0.85	0.40	-0.09	0.23
Treatment group (occupation)	0.05	0.08	0.03	0.62	0.53	-0.11	0.20
Quizzes_children_post (= Correct answer to quiz corresponding to the information in post-phase)	0.09	0.04	0.06	2.13	0.03	0.01	0.17
Q4_children_pre (Children's SESRA-S score in the pre-phase)	0.01	0.00	0.14	4.92	0.00	0.01	0.02
Q8_children_pre (Children's response to the occupations in the pre-phase)	-0.32	0.02	-0.42	-14.56	0.00	-0.36	-0.28
Girl * Treatment Group (occupations & math)	0.00	0.11	0.00	-0.03	0.97	-0.22	0.21
Girl * Treatment Group (occupations & society)	-0.02	0.11	-0.01	-0.17	0.87	-0.24	0.20
Girl * Treatment Group (occupations)	0.03	0.11	0.01	0.28	0.78	-0.19	0.25

Q9: Learning math

	Unstandardized Coefficients		Standardized Coefficients	t	Sig.	95% Confidence Interval for (B)	
	B	Std. Error	β			Lower bound	Upper bound
(Constant)	0.38	0.16		2.42	0.02	0.07	0.69
Gender_children (= girl)	-0.11	0.08	-0.07	-1.35	0.18	-0.28	0.05
Treatment group (occupations & math)	0.17	0.08	0.09	2.02	0.04	0.00	0.33
Treatment group (occupations & society)	-0.01	0.09	-0.01	-0.11	0.91	-0.18	0.16
Treatment group (occupation)	0.10	0.08	0.06	1.21	0.23	-0.06	0.26
Quizzes_children_post (= Correct answer to quiz corresponding to the information in post-phase)	0.16	0.04	0.10	3.48	0.00	0.07	0.24
Q4_children_pre (Children's SESRA-S score in the pre-phase)	0.02	0.00	0.18	6.65	0.00	0.01	0.02
Q9_children_pre (Children's response to learning math in the pre-phase)	-0.39	0.02	-0.47	-17.28	0.00	-0.43	-0.34
Girl * Treatment Group (occupations & math)	0.02	0.12	0.01	0.20	0.84	-0.21	0.25
Girl * Treatment Group (occupations & society)	0.23	0.12	0.10	2.00	0.05	0.00	0.46
Girl * Treatment Group (occupations)	0.00	0.12	0.00	0.02	0.99	-0.23	0.23

Results of parents:

Q3: Motivation to encourage their children to choose STEM

	Unstandardized Coefficients		Standardized Coefficients	t	Sig.	95% Confidence Interval for (B)	
	B	Std. Error	β			Lower bound	Upper bound
(Constant)	-1.65	0.24		-6.86	0.00	-2.13	-1.18
Treatment group (occupations & math)	0.09	0.08	0.05	1.03	0.30	-0.08	0.25
Treatment group (occupations & society)	-0.02	0.09	-0.01	-0.24	0.81	-0.19	0.15
Treatment group (occupation)	0.03	0.08	0.02	0.37	0.71	-0.13	0.19
Gender_parent (= women)	-0.01	0.09	0.00	-0.07	0.95	-0.18	0.16
major (= Science/agriculture/engineering/medicine)	0.15	0.07	0.07	2.33	0.02	0.02	0.28
education (= Those who graduated from university or graduate university)	0.05	0.05	0.03	1.08	0.28	-0.04	0.15
Quizzes_parent_post (= Correct answer to quiz corresponding to the information in post-phase)	0.18	0.04	0.11	3.96	0.00	0.09	0.26
age_p (Age of parents)	0.01	0.00	0.04	1.44	0.15	0.00	0.01
Q4_parents_pre (Parental SESRA-S score in the pre-phase)	0.00	0.00	0.05	1.84	0.07	0.00	0.01
Q3_parents_pre (Parental motivation to encourage their children to choose STEM in the pre-phase)	0.36	0.02	0.49	17.82	0.00	0.32	0.40
Women * Treatment Group (occupations & math)	0.08	0.12	0.04	0.70	0.49	-0.15	0.31
Women * Treatment Group (occupations & society)	0.09	0.12	0.04	0.73	0.47	-0.14	0.31
Women * Treatment Group (occupations)	0.13	0.12	0.06	1.14	0.25	-0.09	0.36

Q4: SESRA-S score

	Unstandardized Coefficients		Standardized Coefficients	t	Sig.	95% Confidence Interval for (B)	
	B	Std. Error	β			Lower bound	Upper bound
(Constant)	2.72	1.59		1.71	0.09	-0.40	5.84
Treatment group (occupations & math)	0.62	0.58	0.06	1.07	0.29	-0.52	1.75
Treatment group (occupations & society)	0.55	0.59	0.05	0.93	0.35	-0.61	1.70
Treatment group (occupation)	0.65	0.57	0.06	1.15	0.25	-0.46	1.76
Gender_parent (= women)	1.03	0.59	0.11	1.74	0.08	-0.13	2.19
major (= Science/agriculture/engineering/medicine)	-0.86	0.45	-0.06	-1.91	0.06	-1.74	0.02
education (= Those who graduated from university or graduate university)	1.35	0.33	0.14	4.04	0.00	0.69	2.00
Quizzes_parent_post (= Correct answer to quiz corresponding to the information in post-phase)	0.62	0.30	0.06	2.05	0.04	0.03	1.21
age_p (Age of parents)	0.06	0.03	0.07	2.26	0.02	0.01	0.12
Q4_parents_pre (Parental SESRA-S score in the pre-phase)	-0.15	0.02	-0.27	-9.12	0.00	-0.18	-0.12
Women * Treatment Group (occupations & math)	0.87	0.79	0.06	1.10	0.27	-0.68	2.42
Women * Treatment Group (occupations & society)	0.01	0.79	0.00	0.01	0.99	-1.55	1.57
Women * Treatment Group (occupations)	-0.31	0.79	-0.02	-0.39	0.69	-1.86	1.24

Q5: Non-stereotypical view of education

	Unstandardized Coefficients		Standardized Coefficients	t	Sig.	95% Confidence Interval for (B)	
	B	Std. Error	β			Lower bound	Upper bound
(Constant)	0.22	0.27		0.81	0.42	-0.31	0.74
Treatment group (occupations & math)	0.03	0.10	0.02	0.33	0.74	-0.16	0.22
Treatment group (occupations & society)	0.04	0.10	0.02	0.44	0.66	-0.15	0.24
Treatment group (occupation)	0.00	0.10	0.00	0.00	1.00	-0.19	0.19
Gender_parent (= women)	0.02	0.10	0.01	0.23	0.82	-0.17	0.22
major (= Science/agriculture/engineering/medicine)	-0.11	0.08	-0.05	-1.50	0.13	-0.26	0.03
education (= Those who graduated from university or graduate university)	0.11	0.06	0.06	2.01	0.05	0.00	0.22
Quizzes_parent_post (= Correct answer to quiz corresponding to the information in post-phase)	0.04	0.05	0.02	0.86	0.39	-0.06	0.14
age_p (Age of parents)	0.00	0.00	0.03	0.86	0.39	-0.01	0.01
Q4_parents_pre (Parental SESRA-S score in the pre-phase)	0.03	0.00	0.25	7.73	0.00	0.02	0.03
Q5_parents_pre (Parental stereotypical view of education in the pre-phase)	-0.54	0.03	-0.61	-18.65	0.00	-0.60	-0.48
Women * Treatment Group (occupations & math)	0.05	0.13	0.02	0.35	0.73	-0.21	0.31
Women * Treatment Group (occupations & society)	0.08	0.13	0.03	0.61	0.54	-0.18	0.35
Women * Treatment Group (occupations)	0.07	0.13	0.02	0.49	0.62	-0.20	0.33

Q6: Non-stereotypical view of math skills

	Unstandardized Coefficients		Standardized Coefficients	t	Sig.	95% Confidence Interval for (B)	
	B	Std. Error	β			Lower bound	Upper bound
(Constant)	0.45	0.27		1.67	0.09	-0.08	0.98
Treatment group (occupations & math)	0.06	0.10	0.03	0.59	0.55	-0.13	0.25
Treatment group (occupations & society)	0.00	0.10	0.00	0.00	1.00	-0.20	0.20
Treatment group (occupation)	-0.03	0.10	-0.02	-0.33	0.74	-0.22	0.16
Gender_parent (= women)	-0.04	0.10	-0.02	-0.36	0.72	-0.23	0.16
major (= Science/agriculture/engineering/medicine)	-0.11	0.08	-0.04	-1.45	0.15	-0.26	0.04
education (= Those who graduated from university or graduate university)	0.17	0.06	0.09	2.97	0.00	0.06	0.28
Quizzes_parent_post (= Correct answer to quiz corresponding to the information in post-phase)	0.10	0.05	0.05	1.89	0.06	0.00	0.20
age_p (Age of parents)	0.00	0.00	0.00	0.17	0.87	-0.01	0.01
Q4_parents_pre (Parental SESRA-S score in the pre-phase)	0.03	0.00	0.25	8.46	0.00	0.02	0.03
Q6_parents_pre (Parental stereotypical view of math skills in the pre-phase)	-0.57	0.03	-0.60	-20.39	0.00	-0.63	-0.52
Women * Treatment Group (occupations & math)	0.20	0.13	0.07	1.48	0.14	-0.07	0.46
Women * Treatment Group (occupations & society)	0.02	0.14	0.01	0.15	0.88	-0.25	0.29
Women * Treatment Group (occupations)	-0.04	0.13	-0.01	-0.28	0.78	-0.30	0.23

Q7: Non-stereotypical view of women’s intellect

	Unstandardized Coefficients		Standardized Coefficients	t	Sig.	95% Confidence Interval for (B)	
	B	Std. Error	β			Lower bound	Upper bound
(Constant)	0.93	0.26		3.57	0.00	0.42	1.45
Treatment group (occupations & math)	-0.13	0.09	-0.07	-1.42	0.16	-0.32	0.05
Treatment group (occupations & society)	-0.19	0.10	-0.10	-2.03	0.04	-0.38	-0.01
Treatment group (occupation)	-0.11	0.09	-0.06	-1.24	0.22	-0.29	0.07
Gender_parent (= women)	-0.01	0.10	-0.01	-0.10	0.92	-0.20	0.18
major (= Science/agriculture/engineering/medicine)	0.01	0.07	0.01	0.19	0.85	-0.13	0.16
education (= Those who graduated from university or graduate university)	0.07	0.05	0.04	1.27	0.20	-0.04	0.18
Quizzes_parent_post (= Correct answer to quiz corresponding to the information in post-phase)	0.12	0.05	0.07	2.41	0.02	0.02	0.21
age_p (Age of parents)	0.00	0.00	0.00	0.08	0.94	-0.01	0.01
Q4_parents_pre (Parental SESRA-S score in the pre-phase)	0.00	0.00	0.04	1.23	0.22	0.00	0.01
Q7_parents_pre (Parental stereotypical view of women's intellect in the pre phase)	-0.34	0.03	-0.38	-12.91	0.00	-0.39	-0.29
Women * Treatment Group (occupations & math)	0.15	0.13	0.06	1.14	0.26	-0.11	0.40
Women * Treatment Group (occupations & society)	0.14	0.13	0.06	1.08	0.28	-0.11	0.39
Women * Treatment Group (occupations)	0.19	0.13	0.08	1.45	0.15	-0.06	0.44
