# Supplementary material for: Effect of providing gender equality information on students’ motivations to choose STEM
Source: PLoS One. 2021 Jun 23;16(6):e0252710. doi: 10.1371/journal.pone.0252710 (PMC8221466; doi:10.1371/journal.pone.0252710)
Supplement: S4 Table — (PDF) [file pone.0252710.s006.pdf]

S4 Table. Full model of third analysis.

Results of children:

Q1: Motivation to choose STEM

	Unstandardized Coefficients		Standardized Coefficients	t	Sig.	95% Confidence Interval for (B)	
	B	Std. Error	β			Lower bound	Upper bound
(Constant)	0.762	0.173		4.417	0.000	0.424	1.101
Gender_children (= girl)	-0.073	0.042	-0.050	-1.754	0.080	-0.156	0.009
Treatment group (occupations & math)	0.103	0.093	0.060	1.104	0.270	-0.080	0.286
Treatment group (occupations & society)	0.040	0.090	0.023	0.446	0.656	-0.137	0.218
Treatment group (occupation)	-0.051	0.097	-0.030	-0.522	0.602	-0.242	0.140
Quizzes_children_post (= Correct answer to quiz corresponding to the information in post-phase)	-0.011	0.092	-0.007	-0.121	0.904	-0.191	0.169
Q4_children_pre (Children's SESRA-S score in the pre-phase)	0.005	0.003	0.055	1.970	0.049	0.000	0.011
Q1_children_pre (Children's motivation to choose STEM in the pre-phase)	-0.308	0.022	-0.406	-14.300	0.000	-0.351	-0.266
Correct answer to quiz * Treatment Group (occupations & math)	0.184	0.124	0.074	1.484	0.138	-0.059	0.427
Correct answer to quiz * Treatment Group (occupations & society)	0.185	0.134	0.058	1.387	0.166	-0.077	0.448
Correct answer to quiz * Treatment Group (occupations)	0.280	0.123	0.128	2.285	0.022	0.040	0.521

Q2: Motivation to participate in STEM events

	Unstandardized Coefficients		Standardized Coefficients	t	Sig.	95% Confidence Interval for (B)	
	B	Std. Error	β			Lower bound	Upper bound
(Constant)	0.289	0.150		1.921	0.055	-0.006	0.584
Gender_children (= girl)	-0.019	0.038	-0.015	-0.514	0.607	-0.093	0.054
Treatment group (occupations & math)	0.160	0.084	0.108	1.899	0.058	-0.005	0.326
Treatment group (occupations & society)	0.018	0.082	0.012	0.219	0.827	-0.143	0.179
Treatment group (occupation)	0.012	0.088	0.008	0.132	0.895	-0.161	0.184
Quizzes_children_post (= Correct answer to quiz corresponding to the information in post-phase)	0.080	0.083	0.062	0.962	0.336	-0.083	0.243
Q4_children_pre (Children's SESRA-S score in the pre-phase)	0.005	0.002	0.060	2.058	0.040	0.000	0.010
Q2_children_pre (Children's motivation to participate in "STEM-events" in the pre-phase)	-0.197	0.018	-0.317	-10.863	0.000	-0.232	-0.161
Correct answer to quiz * Treatment Group (occupations & math)	-0.025	0.112	-0.011	-0.219	0.827	-0.245	0.196
Correct answer to quiz * Treatment Group (occupations & society)	0.042	0.121	0.015	0.346	0.730	-0.196	0.280
Correct answer to quiz * Treatment Group (occupations)	0.073	0.111	0.038	0.658	0.511	-0.145	0.291

Q4: SESRA-S score

	Unstandardized Coefficients		Standardized Coefficients	t	Sig.	95% Confidence Interval for (B)	
	B	Std. Error	β			Lower bound	Upper bound
(Constant)	3.143	0.872		3.603	0.000	1.431	4.854
Gender_children (= girl)	0.104	0.233	0.013	0.448	0.654	-0.352	0.561
Treatment group (occupations & math)	1.095	0.528	0.123	2.075	0.038	0.060	2.131
Treatment group (occupations & society)	0.844	0.512	0.094	1.649	0.099	-0.160	1.848
Treatment group (occupation)	0.863	0.550	0.097	1.568	0.117	-0.217	1.942
Quizzes_children_post (= Correct answer to quiz corresponding to the information in post-phase)	1.020	0.519	0.131	1.967	0.049	0.002	2.038
Q4_children_pre (Children's SESRA-S score in the pre-phase)	-0.080	0.015	-0.157	-5.147	0.000	-0.110	-0.049
Correct answer to quiz * Treatment Group (occupations & math)	-0.375	0.702	-0.029	-0.534	0.593	-1.751	1.002
Correct answer to quiz * Treatment Group (occupations & society)	0.622	0.758	0.037	0.822	0.412	-0.864	2.109
Correct answer to quiz * Treatment Group (occupations)	-0.482	0.693	-0.042	-0.695	0.487	-1.842	0.878

Q5: Non-stereotypical view of education

	Unstandardized Coefficients		Standardized Coefficients	t	Sig.	95% Confidence Interval for (B)	
	B	Std. Error	β			Lower bound	Upper bound
(Constant)	0.250	0.151		1.656	0.098	-0.046	0.547
Gender_children (= girl)	0.016	0.040	0.011	0.396	0.692	-0.063	0.095
Treatment group (occupations & math)	-0.068	0.091	-0.040	-0.745	0.457	-0.247	0.111
Treatment group (occupations & society)	-0.094	0.089	-0.054	-1.059	0.290	-0.268	0.080
Treatment group (occupation)	-0.009	0.095	-0.005	-0.096	0.924	-0.196	0.178
Quizzes_children_post (= Correct answer to quiz corresponding to the information in post-phase)	-0.040	0.090	-0.027	-0.447	0.655	-0.217	0.136
Q4_children_pre (Children's SESRA-S score in the pre-phase)	0.031	0.003	0.311	9.053	0.000	0.024	0.037
Q5_children_pre (Children's stereotypical view of education in the pre-phase)	-0.513	0.029	-0.602	-17.649	0.000	-0.570	-0.456
Correct answer to quiz * Treatment Group (occupations & math)	0.033	0.121	0.013	0.275	0.783	-0.205	0.272
Correct answer to quiz * Treatment Group (occupations & society)	0.202	0.131	0.062	1.539	0.124	-0.056	0.460
Correct answer to quiz * Treatment Group (occupations)	0.063	0.120	0.029	0.527	0.599	-0.173	0.299

Q6: Non-stereotypical view of math skills

	Unstandardized Coefficients		Standardized Coefficients	t	Sig.	95% Confidence Interval for (B)	
	B	Std. Error	β			Lower bound	Upper bound
(Constant)	0.166	0.155		1.070	0.285	-0.139	0.471
Gender_children (= girl)	-0.051	0.041	-0.034	-1.232	0.218	-0.132	0.030
Treatment group (occupations & math)	0.023	0.094	0.013	0.249	0.803	-0.161	0.208
Treatment group (occupations & society)	-0.087	0.091	-0.050	-0.957	0.339	-0.266	0.092
Treatment group (occupation)	0.031	0.098	0.018	0.313	0.754	-0.161	0.223
Quizzes_children_post (= Correct answer to quiz corresponding to the information in post-phase)	0.016	0.092	0.010	0.169	0.866	-0.166	0.197
Q4_children_pre (Children's SESRA-S score in the pre-phase)	0.028	0.003	0.279	8.445	0.000	0.021	0.034
Q6_children_pre (Children's stereotypical view of math skills in the pre-phase)	-0.445	0.027	-0.532	-16.207	0.000	-0.499	-0.391
Correct answer to quiz * Treatment Group (occupations & math)	0.114	0.125	0.045	0.910	0.363	-0.132	0.359
Correct answer to quiz * Treatment Group (occupations & society)	0.155	0.135	0.048	1.151	0.250	-0.109	0.420
Correct answer to quiz * Treatment Group (occupations)	-0.194	0.123	-0.087	-1.570	0.117	-0.436	0.048

Q7: Non-stereotypical view of women's intellect

	Unstandardized Coefficients		Standardized Coefficients	t	Sig.	95% Confidence Interval for (B)	
	B	Std. Error	β			Lower bound	Upper bound
(Constant)	0.494	0.159		3.114	0.002	0.183	0.806
Gender_children (= girl)	0.006	0.038	0.004	0.145	0.885	-0.069	0.080
Treatment group (occupations & math)	0.059	0.087	0.039	0.678	0.498	-0.111	0.229
Treatment group (occupations & society)	0.003	0.084	0.002	0.034	0.973	-0.162	0.168
Treatment group (occupation)	-0.046	0.090	-0.030	-0.511	0.610	-0.223	0.131
Quizzes_children_post (= Correct answer to quiz corresponding to the information in post-phase)	0.051	0.085	0.038	0.597	0.551	-0.116	0.218
Q4_children_pre (Children's SESRA-S score in the pre-phase)	0.008	0.003	0.087	2.976	0.003	0.003	0.013
Q7_children_pre (Children's stereotypical view of women's intellect in the pre phase)	-0.261	0.023	-0.335	-11.546	0.000	-0.305	-0.217
Correct answer to quiz * Treatment Group (occupations & math)	-0.001	0.115	0.000	-0.007	0.994	-0.227	0.226
Correct answer to quiz * Treatment Group (occupations & society)	-0.094	0.124	-0.033	-0.754	0.451	-0.338	0.150
Correct answer to quiz * Treatment Group (occupations)	0.090	0.114	0.046	0.792	0.429	-0.133	0.314

Q8: Occupations

	Unstandardized Coefficients		Standardized Coefficients	t	Sig.	95% Confidence Interval for (B)	
	B	Std. Error	β			Lower bound	Upper bound
(Constant)	0.477	0.159		3.001	0.003	0.165	0.788
Gender_children (= girl)	-0.023	0.039	-0.017	-0.589	0.556	-0.100	0.054
Treatment group (occupations & math)	0.064	0.089	0.040	0.719	0.472	-0.110	0.237
Treatment group (occupations & society)	0.076	0.086	0.047	0.880	0.379	-0.093	0.244
Treatment group (occupation)	0.036	0.092	0.023	0.394	0.694	-0.145	0.218
Quizzes_children_post (= Correct answer to quiz corresponding to the information in post-phase)	0.088	0.087	0.063	1.015	0.310	-0.082	0.259
Q4_children_pre (Children's SESRA-S score in the pre-phase)	0.013	0.003	0.143	4.945	0.000	0.008	0.018
Q8_children_pre (Children's response to the occupations in the pre-phase)	-0.322	0.022	-0.419	-14.601	0.000	-0.366	-0.279
Correct answer to quiz * Treatment Group (occupations & math)	0.005	0.118	0.002	0.045	0.964	-0.226	0.236
Correct answer to quiz * Treatment Group (occupations & society)	-0.075	0.127	-0.025	-0.586	0.558	-0.324	0.175
Correct answer to quiz * Treatment Group (occupations)	0.053	0.117	0.026	0.458	0.647	-0.175	0.282

Q9: Learning math

	Unstandardized Coefficients		Standardized Coefficients	t	Sig.	95% Confidence Interval for (B)	
	B	Std. Error	β			Lower bound	Upper bound
(Constant)	0.535	0.166		3.222	0.001	0.209	0.861
Gender_children (= girl)	-0.049	0.041	-0.032	-1.186	0.236	-0.130	0.032
Treatment group (occupations & math)	0.010	0.094	0.006	0.107	0.915	-0.174	0.194
Treatment group (occupations & society)	-0.055	0.091	-0.031	-0.604	0.546	-0.233	0.123
Treatment group (occupation)	-0.085	0.098	-0.048	-0.871	0.384	-0.276	0.106
Quizzes_children_post (= Correct answer to quiz corresponding to the information in post-phase)	-0.051	0.092	-0.033	-0.557	0.577	-0.232	0.129
Q4_children_pre (Children's SESRA-S score in the pre-phase)	0.018	0.003	0.176	6.466	0.000	0.012	0.023
Q9_children_pre (Children's response to learning math in the pre-phase)	-0.390	0.022	-0.471	-17.459	0.000	-0.434	-0.346
Correct answer to quiz * Treatment Group (occupations & math)	0.261	0.124	0.101	2.101	0.036	0.017	0.506
Correct answer to quiz * Treatment Group (occupations & society)	0.269	0.134	0.080	2.001	0.046	0.005	0.533
Correct answer to quiz * Treatment Group (occupations)	0.279	0.123	0.122	2.270	0.023	0.038	0.521

Results of parents:

Q3: Motivation to encourage their children to choose STEM

	Unstandardized Coefficients		Standardized Coefficients	t	Sig.	95% Confidence Interval for (B)	
	B	Std. Error	β			Lower bound	Upper bound
(Constant)	0.642	0.245		2.618	0.009	0.161	1.123
Treatment group (occupations & math)	-0.070	0.099	-0.040	-0.709	0.479	-0.265	0.124
Treatment group (occupations & society)	-0.122	0.097	-0.069	-1.264	0.206	-0.312	0.067
Treatment group (occupation)	-0.061	0.104	-0.035	-0.586	0.558	-0.264	0.143
Gender_parent (= women)	0.066	0.047	0.043	1.396	0.163	-0.027	0.159
Major (= Science/agriculture/engineering/medicine)	0.152	0.066	0.072	2.316	0.021	0.023	0.281
Education (= Those who graduated from university or graduate university)	0.052	0.049	0.034	1.066	0.286	-0.044	0.149
Quizzes_parent_post (= Correct answer to quiz corresponding to the information in post-phase)	-0.017	0.096	-0.011	-0.177	0.860	-0.206	0.172
Age_p (age of parents)	0.006	0.004	0.044	1.469	0.142	-0.002	0.014
Q4_parents_pre (Parental SESRA-S score in the pre-phase)	0.004	0.002	0.048	1.780	0.075	0.000	0.009
Q3_parents_pre (Parental motivation to encourage their children to choose STEM in the pre-phase)	-0.365	0.020	-0.491	-17.931	0.000	-0.405	-0.325
Correct answer to quiz * Treatment Group (occupations & math)	0.319	0.126	0.128	2.523	0.012	0.071	0.566
Correct answer to quiz * Treatment Group (occupations & society)	0.185	0.133	0.060	1.383	0.167	-0.077	0.447
Correct answer to quiz * Treatment Group (occupations)	0.215	0.126	0.098	1.709	0.088	-0.032	0.463

Q4: SESRA-S score

	Unstandardized Coefficients		Standardized Coefficients	t	Sig.	95% Confidence Interval for (B)	
	B	Std. Error	β			Lower bound	Upper bound
(Constant)	2.714	1.623		1.673	0.095	-0.470	5.899
Treatment group (occupations & math)	0.819	0.678	0.075	1.208	0.227	-0.511	2.149
Treatment group (occupations & society)	0.474	0.659	0.043	0.719	0.472	-0.820	1.768
Treatment group (occupation)	1.091	0.708	0.099	1.540	0.124	-0.299	2.481
Gender_parent (= women)	1.143	0.324	0.119	3.528	0.000	0.507	1.779
Major (= Science/agriculture/engineering/medicine)	-0.861	0.448	-0.065	-1.922	0.055	-1.740	0.018
Education (= Those who graduated from university or graduate university)	1.363	0.332	0.142	4.108	0.000	0.712	2.014
Quizzes_parent_post (= Correct answer to quiz corresponding to the information in post-phase)	0.669	0.658	0.070	1.016	0.310	-0.622	1.959
Age_p (age of parents)	0.060	0.028	0.071	2.199	0.028	0.007	0.114
Q4_parents_pre (Parental SESRA-S score in the pre-phase)	-0.150	0.016	-0.272	-9.143	0.000	-0.182	-0.118
Correct answer to quiz * Treatment Group (occupations & math)	0.630	0.862	0.041	0.730	0.465	-1.062	2.322
Correct answer to quiz * Treatment Group (occupations & society)	0.396	0.911	0.021	0.434	0.664	-1.392	2.184
Correct answer to quiz * Treatment Group (occupations)	-1.041	0.861	-0.076	-1.209	0.227	-2.731	0.649

Q5: Non-stereotypical view of education

	Unstandardized Coefficients		Standardized Coefficients	t	Sig.	95% Confidence Interval for (B)	
	B	Std. Error	β			Lower bound	Upper bound
(Constant)	0.108	0.274		0.395	0.693	-0.429	0.645
Treatment group (occupations & math)	0.148	0.114	0.072	1.294	0.196	-0.076	0.372
Treatment group (occupations & society)	0.153	0.111	0.074	1.376	0.169	-0.065	0.371
Treatment group (occupation)	0.164	0.119	0.080	1.378	0.168	-0.070	0.399
Gender_parent (= women)	0.073	0.055	0.041	1.329	0.184	-0.035	0.180
Major (= Science/agriculture/engineering/medicine)	-0.113	0.075	-0.046	-1.492	0.136	-0.261	0.036
Education (= Those who graduated from university or graduate university)	0.115	0.056	0.064	2.053	0.040	0.005	0.226
Quizzes_parent_post (= Correct answer to quiz corresponding to the information in post-phase)	0.150	0.111	0.084	1.357	0.175	-0.067	0.368
Age_p (age of parents)	0.004	0.005	0.025	0.860	0.390	-0.005	0.013
Q4_parents_pre (Parental SESRA-S score in the pre-phase)	0.026	0.003	0.254	7.739	0.000	0.019	0.033
Q5_parents_pre (Parental stereotypical view of Education in the pre-phase)	-0.541	0.029	-0.608	-18.631	0.000	-0.598	-0.484
Correct answer to quiz * Treatment Group (occupations & math)	-0.132	0.145	-0.046	-0.907	0.365	-0.417	0.153
Correct answer to quiz * Treatment Group (occupations & society)	-0.055	0.154	-0.015	-0.359	0.719	-0.356	0.246
Correct answer to quiz * Treatment Group (occupations)	-0.195	0.145	-0.077	-1.346	0.178	-0.480	0.089

Q6: Non-stereotypical view of math skills

	Unstandardized Coefficients		Standardized Coefficients	t	Sig.	95% Confidence Interval for (B)	
	B	Std. Error	β			Lower bound	Upper bound
(Constant)	0.377	0.276		1.365	0.173	-0.165	0.920
Treatment group (occupations & math)	0.165	0.115	0.078	1.426	0.154	-0.062	0.391
Treatment group (occupations & society)	0.080	0.112	0.038	0.714	0.475	-0.140	0.301
Treatment group (occupation)	0.105	0.121	0.050	0.868	0.386	-0.132	0.342
Gender_parent (= women)	0.006	0.055	0.003	0.100	0.920	-0.103	0.114
Major (= Science/agriculture/engineering/medicine)	-0.111	0.076	-0.044	-1.456	0.146	-0.261	0.039
Education (= Those who graduated from university or graduate university)	0.172	0.057	0.093	3.036	0.002	0.061	0.283
Quizzes_parent_post (= Correct answer to quiz corresponding to the information in post-phase)	0.180	0.112	0.098	1.605	0.109	-0.040	0.400
Age_p (age of parents)	0.001	0.005	0.004	0.128	0.898	-0.009	0.010
Q4_parents_pre (Parental SESRA-S score in the pre-phase)	0.027	0.003	0.256	8.615	0.000	0.021	0.033
Q6_parents_pre (Parental stereotypical view of math skills in the pre-phase)	-0.580	0.028	-0.610	-20.603	0.000	-0.635	-0.525
Correct answer to quiz * Treatment Group (occupations & math)	0.058	0.147	0.019	0.395	0.693	-0.230	0.346
Correct answer to quiz * Treatment Group (occupations & society)	-0.108	0.155	-0.029	-0.694	0.488	-0.412	0.197
Correct answer to quiz * Treatment Group (occupations)	-0.248	0.147	-0.094	-1.685	0.092	-0.536	0.041

Q7: Non-stereotypical view of women's intellect

	Unstandardized Coefficients		Standardized Coefficients	t	Sig.	95% Confidence Interval for (B)	
	B	Std. Error	β			Lower bound	Upper bound
(Constant)	1.022	0.268		3.813	0.000	0.496	1.548
Treatment group (occupations & math)	-0.240	0.110	-0.131	-2.189	0.029	-0.456	-0.025
Treatment group (occupations & society)	-0.279	0.107	-0.151	-2.608	0.009	-0.488	-0.069
Treatment group (occupation)	-0.147	0.115	-0.080	-1.280	0.201	-0.372	0.078
Gender_parent (= women)	0.106	0.053	0.067	2.017	0.044	0.003	0.210
Major (= Science/agriculture/engineering/medicine)	0.010	0.073	0.005	0.138	0.890	-0.133	0.153
Education (= Those who graduated from university or graduate university)	0.071	0.055	0.044	1.298	0.195	-0.036	0.178
Quizzes_parent_post (= Correct answer to quiz corresponding to the information in post-phase)	-0.063	0.107	-0.040	-0.595	0.552	-0.272	0.146
Age_p (age of parents)	0.001	0.004	0.005	0.142	0.887	-0.008	0.009
Q4_parents_pre (Parental SESRA-S score in the pre-phase)	0.003	0.003	0.034	1.165	0.244	-0.002	0.008
Q7_parents_pre (Parental stereotypical view of women's intellect in the pre phase)	-0.343	0.026	-0.383	-13.073	0.000	-0.395	-0.292
Correct answer to quiz * Treatment Group (occupations & math)	0.287	0.140	0.111	2.056	0.040	0.013	0.562
Correct answer to quiz * Treatment Group (occupations & society)	0.248	0.148	0.077	1.683	0.093	-0.041	0.538
Correct answer to quiz * Treatment Group (occupations)	0.164	0.140	0.072	1.172	0.241	-0.110	0.438
